# Simulated dataset of corn response to nitrogen over thousands of fields and multiple years in Illinois

**DOI:** 10.1016/j.dib.2021.107753

**Published:** 2021-12-28

**Authors:** German Mandrini, Sotirios V. Archontoulis, Cameron M. Pittelkow, Taro Mieno, Nicolas F. Martin

**Affiliations:** a Department of Crop Sciences, University of Illinois at Urbana-Champaign, W201 Turner Hall, 1102 S. Goodwin Avenue, Urbana, IL 61801, USA; b Department of Agronomy, Iowa State University, Ames, IA 50011, USA; c Department of Plant Sciences, University of California, Davis, CA 95616 USA; d Department of Agricultural Economics, University of Nebraska-Lincoln, Lincoln, NE 68583 0922, USA

**Keywords:** APSIM, Crop modeling, Maize, Nitrogen leaching, Nitrogen fertilizer

## Abstract

Nitrogen (N) fertilizer recommendations for corn (Zea mays L.) in the US Midwest have been a puzzle for several decades, without agreement among stakeholders for which methodology is the best to balance environmental and economic outcomes. Part of the reason is the lack of long-term data of crop responses to N over multiple fields since trial data is often limited in the number of soils and years it can explore. To overcome this limitation, we designed an analytical platform based on crop simulations run over millions of farming scenarios over extensive geographies. The database was calibrated and validated using data from more than four hundred trials in the region. This dataset can have an important role for research and education in N management, machine leaching, and environmental policy analysis. The calibration and validation procedure provides a framework for future gridded crop model studies. We describe dataset characteristics and provide thorough descriptions of the model setup.

## Specifications Table

**Table:** 

Subject	Agronomy and Crop Sciences
Specific subject area	Crop and N leaching response to N fertilizer
Type of data	R objects
How data were acquired	Weather data from Daymet
	Soil data from SSURGO
	Simulations using APSIM version 7.1
Data format	Raw
	Analyzed
Parameters for data collection	Soil level simulations were performed. Inputs for the crop model were obtained from public available data sets (DAYMET, SSURGO)
Description of data collection	Data were collected for 4270 fields. The fields had a soy-corn rotation from 1989-2018. During the corn year, the field received N rates from 0 to 320 kg/ha, with 10 kg/ha. Yield, N leaching and multiple other variables were obtained as output from APSIM
Data source location	City/Town/Region:Illinois
	Country:US
Data accessibility	Repository Name: Mendeley Data:
	Data identification number: 10.17632/xs5nbm4w55.1
	Direct URL to data: https://data.mendeley.com/datasets/xs5nbm4w55/1
Related research article	Mandrini, C. M. Pittelkow, S. V. Archontoulis, T. Mieno, N. F. Martin, Understanding differences between static and dynamic nitrogen fertilizer tools using simulation modeling, Agricultural Systems 194 (2021) 103275

## Value of the Data

• This datasets provide an enormous amount of calibrated response curves of several variables to increasing N rates in one of the most productive areas in the world
• It can be used in many different types of studies focused on N management, from an agricultural and environmental perspective
• Some possible ideas are comparing different strategies can be N prediction methods, evaluate policies designed to lower N leaching, evaluation of variable rate N applications
• Machine learning researchers can use the datasets for benchmarking the performance of different algorithms for predicting N rates;
• Educators can use the datasets for machine learning problems, statistics, or data mining training.
• The simulation and calibration methodology is innovative and can be used for other simulations, including different crops or areas than the ones shown here

## 1 Data Description

We provide several datasets in this paper used in the research article “Understanding differences between static and dynamic nitrogen fertilizer tools using simulation modeling” [1]. The datasets consist of soil and weather information, the fields’ locations, and the simulations’ output for 4270 fields over 30 years.

### 1.1 Spatial files

• cells_sf: polygons of the 10 x 10 km cells on which the state of Illinois was divided. The id_10 is an identifier of each cell. It also includes the region (South, Central, North), the county, and the average area planted to corn (ha/year) in 2008 to 2019.
• fields_sf: polygons of the 40-ha fields. The id_field (1–4) is the identifier of each field inside a cell. It also contains the id_10, the region, and if a field was used as a trial or evaluation field.
• soils_sf: polygons of the soils inside each field. The mukey is the identifier of each soil. It also contains the id_10, the id_field. Only the three main soils were selected for the simulations, and the column mukey_rank identifies them with a number from 1 to 3 (being 1 the largest and 3 the smallest).

### 1.2 Weather data series

The file weather_historic_dt is a table that describes the weather based on the grid of cells (10 x 10 km) provided by Daymet [2]. The table contains the id_10, the year, the daily temperature (minimum, medium, and maximum), rainfall, and radiation.

### 1.3 Soil information

The file soils_horizons_dt is a table that describes the soils’ layers of each field. The mukey is the identifier of each soil. It includes the water table depth, slope, sand, clay, organic matter, vertical saturated hydraulic conductivity (ksat), lower and upper volumetric water content limit (ll and dul), and ph.

### 1.4 APSIM output

The file yield_curve_soil_dt contains the output of the simulations at the soil level (mukey). The file yield_curve_field_dt includes the output of the simulations at the field level (id_10 and id_field), aggregated considering the area of each of the soils inside a field. A description of the columns is provided (Table 1).

**Table 1:** Database characterization, with identification variables and APSIM output variables.

Variable	Description	Units
region	region identification (1-South, 2-Cental, 3-North)	−
id_10	cell identification number	−
id_field	field identification number (1 to 4)	−
station	trial field (1) or evaluation field (0)	−
year	year of the corn simulation (1989-2018)	−
N_fert	Nitrogen added as fertilizer in v5	kg/ha
Yield	Yield of the corn in with 15% Moisture	kg/ha
L	Total 2-years N leaching during corn and soybean.From April 1^*st*^ year (x) to March 31^*st*^ year (x+2)	N kg/ha
clay_40cm	Clay content (0-20 cm)	%
day_sow	Planting date	Julian date
day_v5	Date when the corn reached v5	Julian date
dul_dep	Drained upper limit (DUL) soil water capacity	mm
esw_pct_v5	Extractable soil water (ESW) at v5	%
LAI_max	maximum LAI achieved by the corn	${\text{m}}^{2}/{\text{m}}^{2}$
lai_v5	Leaf Area Index at v5	${\text{m}}^{2}/{\text{m}}^{2}$
ll15_dep	Crop lower limit soil water capacity	mm
n_0_60cm_v5	Soil N ($NO_{3}$ and $NH_{4}$) from 0 to 60 cm at v5	kg/ha
n_20cm_v5	Soil N ($NO_{3}$ and $NH_{4}$) from 0 to 20 cm at v5	kg/ha
n_40cm_v5	Soil N ($NO_{3}$ and $NH_{4}$) from 0 to 40 cm at v5	kg/ha
n_60cm_v5	Soil N ($NO_{3}$ and $NH_{4}$) from 0 to 60 cm at v5	kg/ha
n_deep_v5	Soil N ($NO_{3}$ and $NH_{4}$) from top to bottom at v5	kg/ha
n_uptake	Total N uptaken by the corn crop during the season	kg/ha
oc_20cm_v5	Soil Organic Carbon at v5 (0-20 cm)	%
oc_40cm_v5	Soil Organic Carbon at v5 (0-40 cm)	%
rad_1	Average solar radiation during first period (1 Jan. to planting)	${\text{MJ/m}}^{2}/\text{day}$
rad_2	Average solar radiation during second period (planting to v5)	${\text{MJ/m}}^{2}/\text{day}$
rad_3	Average solar radiation during third period (v5- R1)	${\text{MJ/m}}^{2}/\text{day}$
rad_4	Average solar radiation during fourth period (R1-R3)	${\text{MJ/m}}^{2}/\text{day}$
rad_5	Average solar radiation during fifth period (R3-R6)	${\text{MJ/m}}^{2}/\text{day}$
rad_6	Average solar radiation during sixth period (harvest-Dec 31)	${\text{MJ/m}}^{2}/\text{day}$
rain_1	Total precipitation during first period (1 Jan. to planting)	mm
rain_2	Total precipitation during second period (planting to v5)	mm
rain_3	Total precipitation during third period (v5-R1)	mm
rain_4	Total precipitation during fourth period (R1-R3)	mm
rain_5	Total precipitation during fifth period (R3-R6)	mm
rain_6	Total precipitation during sixth period (darvest-Dec 31)	mm
restriction	Soil restriction	mm
sand_40cm	Sand content (0-20 cm)	%
surfaceom_wt_v5	Surface residue weight at v5	kg/ha
sw_dep_v5	Soil water content at v5	mm
swdef_expan_fw	Mean water stress on expansion around flowering (APSIM corn stages 6 to 8)	0-1
swdef_pheno_fw	Mean water stress on phenology around flowering (APSIM corn stages 6 to 8)	0-1
swdef_photo_fw	Mean water stress on photosinthesis around flowering (APSIM corn stages 6 to 8)	0-1
tmean_1	Average air temperature during first period (1 Jan. to planting)	${}^{\circ }$C
tmean_2	Average air temperature during second period (planting to v5)	${}^{\circ }$C
tmean_3	Average air temperature during third period (v5-R1)	${}^{\circ }$C
tmean_4	Average air temperature during fourth period (R1-R3)	${}^{\circ }$C
tmean_5	Average air temperature during fifth period (R3-R6)	${}^{\circ }$C
tmean_6	Average air temperature during sixth period (harvest-Dec 31)	${}^{\circ }$C
whc	Water holding capacity	mm
Y_corn_lt_avg	Mean yield at EONR (for the other 29 years)	kg/ha
Y_soy	Yield of soy with 13% Moisture (year+1)	kg/ha
Yld_lt_avg	Mean yield at EONR (for the other 29 years)	kg/ha

### 1.5 Tutorial script

This R Markdown file shows an example of how the data can be used for education or research purposes. It loads the needed files on the script and trains a static and dynamic model with the research fields and the first 15 years of data. Then, it evaluates both models in the evaluation fields in the following 15 years. It finally shows the economic and environmental value of dynamic recommendations on a map.

## 2 Experimental Design, Materials and Methods

### 2.1 The APSIM software

The main goal of the simulations is to obtain information on corn response to increasing N rates for a broad combination of weather and soil conditions. For that, we built upon the simulations presented on [3] with the following adjustments: no-spin up simulations were run, and initial N in the soil was set randomly among a reasonable range, simulations were updated to include a water table when needed, and hybrid parameters were modified to match corn yields per region better. More details about these adjustments are explained later in detail in this work, and the validation results will be presented.

The simulations were conducted using the Agricultural Production Systems sIMulator (APSIM) [4] version 7.10 to generate and calibrate a database for thousands of fields in Illinois. A total of more than 6 million simulations were executed using the Illinois Campus Cluster, a computing resource supported by the University of Illinois at Urbana-Champaign. It is operated by the Illinois Campus Cluster Program (ICCP) in conjunction with the National Center for Supercomputing Applications (NCSA).

The APSIM simulation framework reproduces various processes related to the crop-soil system and environmental factors, allowing for the interaction of these processes in daily simulations. Related processes are grouped into modules. In this study, we used the maize and soybean modules to simulate crop growth, SoilWat for water balance simulation, SurfaceOM for simulation of residue decomposition, and soilN for simulation of soil carbon and N cycle.

In order to reflect the conditions of the Midwest, we modified the soybean and SurfaceOM models according to [5] and the maize module according to [6]. These parameters were guided by calibration and literature and allowed APSIM to better represent the Midwest’s growing conditions, as evidenced in the results.

### 2.2 Input files creation

**Fig. 1:**
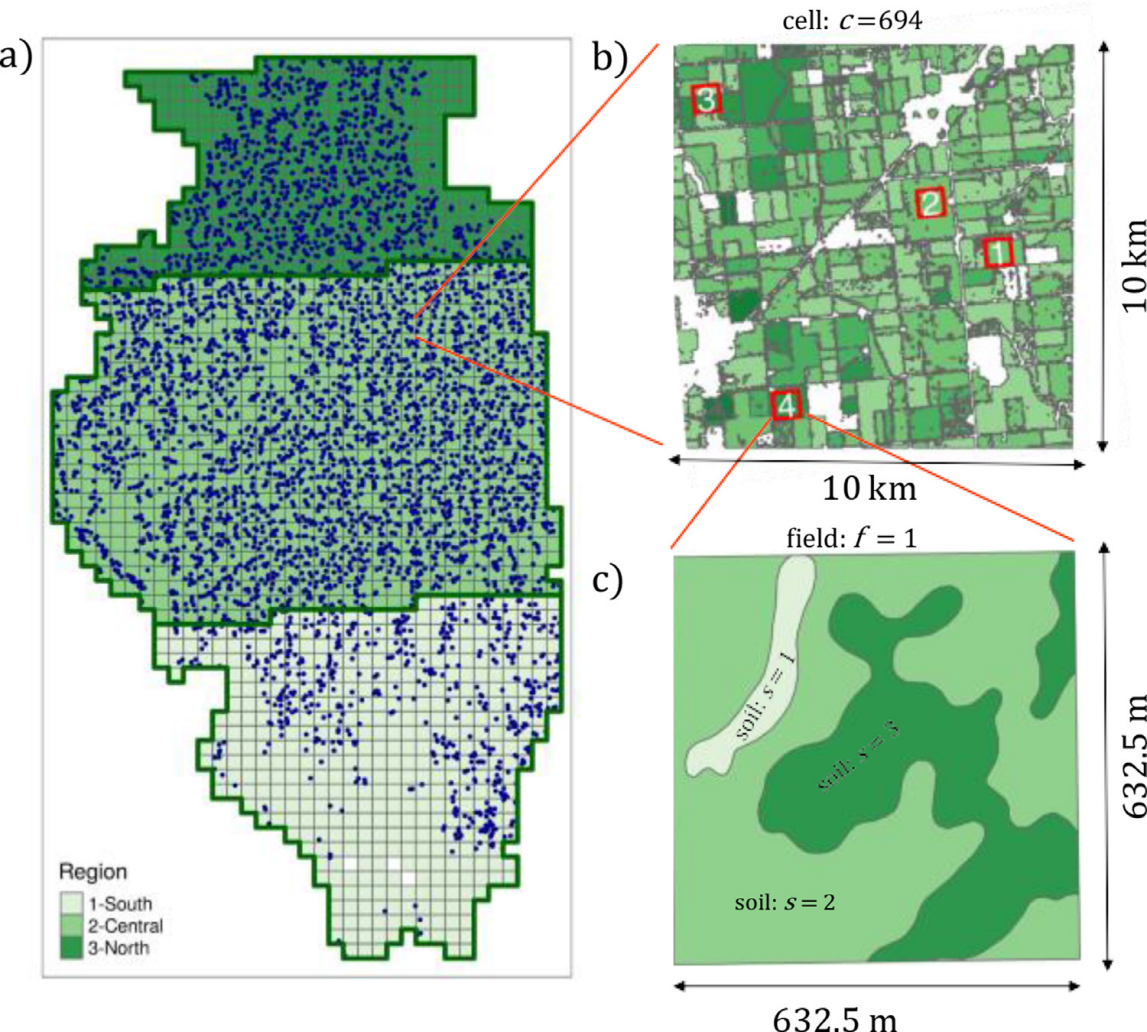
(a) Map of Illinois, showing the grid of cells, the three regions, and the 4270 fields (blue dots). (b) One cell with four fields randomly placed in the target area. (c) Soils obtained for one of those fields.

To guide the simulations and represent the soil and weather variation seen in the region, we divided the state of Illinois into a grid of 10 x 10 km “cells” (Fig. 1a). Four 40-ha square, artificially determined “fields” were then located within each cell (Fig. 1b). These fields were selected from within areas that had been planted to corn for at least five years between 2008 and 2019 according to the USDA Crop Frequency Layer (target area). The fields did not follow actual field boundaries and were allowed to contain parts of multiple actual fields. For cells that did not contain enough target area to create four fields, the maximum possible number of fields were selected, even if this equaled less than four fields. This process yielded 4270 fields, and provided a strong foundation for simulations by increasing simulations in areas with high contribution to crop production and limiting simulations in areas with a low contribution to crop production.

Simulations were conducted using the historical weather for the period 1989-2019. The weather information was obtained from DAYMET [2], using the R “daymetr” package [7]. The weather information consisted of daily radiation, temperature (minimum and maximum), and precipitation, and the same weather was used for all fields in a particular cell.

The makeup of the soil on each of the fields was determined using the USDA Soil Survey Geographic Database (SSURGO) [8]. For this, we first obtain the soil map unit polygons (Fig. 1 c). These polygons provided the identifier of the soil (known as mukey) and the area. For each soil, we obtained profile information by searching gSSURGO using the R soilDB2 package [9], and transformed it into APSIM parameters following the methodology found in [10]. If SSURGO (through the mukey) indicated the presence of several soils in a particular field, the three largest ones were chosen for the simulation, and additional soils from the field were distributed proportionally among the main three. A maximum, constant soil depth of 200 cm was applied to all fields. The Root exploration factor (xF) in the south region was set to 0.1 for soil layers below 1.5 m, slowing the root’s front advance, while in the other two regions, it was set to 1 for all the layers. The adjusted FBiom and FInert are presented in Table 2.

**Table 2:** Soil parameters for each region in the state of Illinois.

	South		Central		North	
Depth (cm)	Fbiom	Finert	Fbiom	Finert	Fbiom	Finert
0-5	0.03	0.5	0.07	0.45	0.08	0.4
5-10	0.025	0.55	0.06	0.5	0.07	0.45
10-15	0.02	0.6	0.05	0.55	0.06	0.47
15-20	0.015	0.75	0.035	0.6	0.05	0.48
20-40	0.015	0.8	0.015	0.65	0.04	0.49
40-60	0.01	0.85	0.01	0.7	0.02	0.5
60-80	0.005	0.9	0.005	0.75	0.01	0.55
80-100	0.001	0.95	0.005	0.8	0.01	0.75
100-150	0.001	0.97	0.001	0.92	0.005	0.9
150-200	0.001	0.99	0.001	0.98	0.001	0.98

The creation of input files also requires setting for the initial conditions from which simulations are started. The initial N concentration of the soil was randomly selected between 1 and 40 kg/ha of N–${\text{NO}}_{3}$. This range of N concentrations was decided by performing a 9-year “spin-up” period test on sampled fields to determine the distribution of possible initial N rates. Soil water was set to field capacity. The initial soybean surface residue was 2000 kg/ha with a C:N ratio of 20. The initial root weight was set to 1000 kg/ha, with a C:N ratio of 13. The organic carbon was obtained from SSURGO data, and the calibration procedure obtained the fractions of the different organic pools (FBiom and FInert), explained later.

**Fig. 2:**
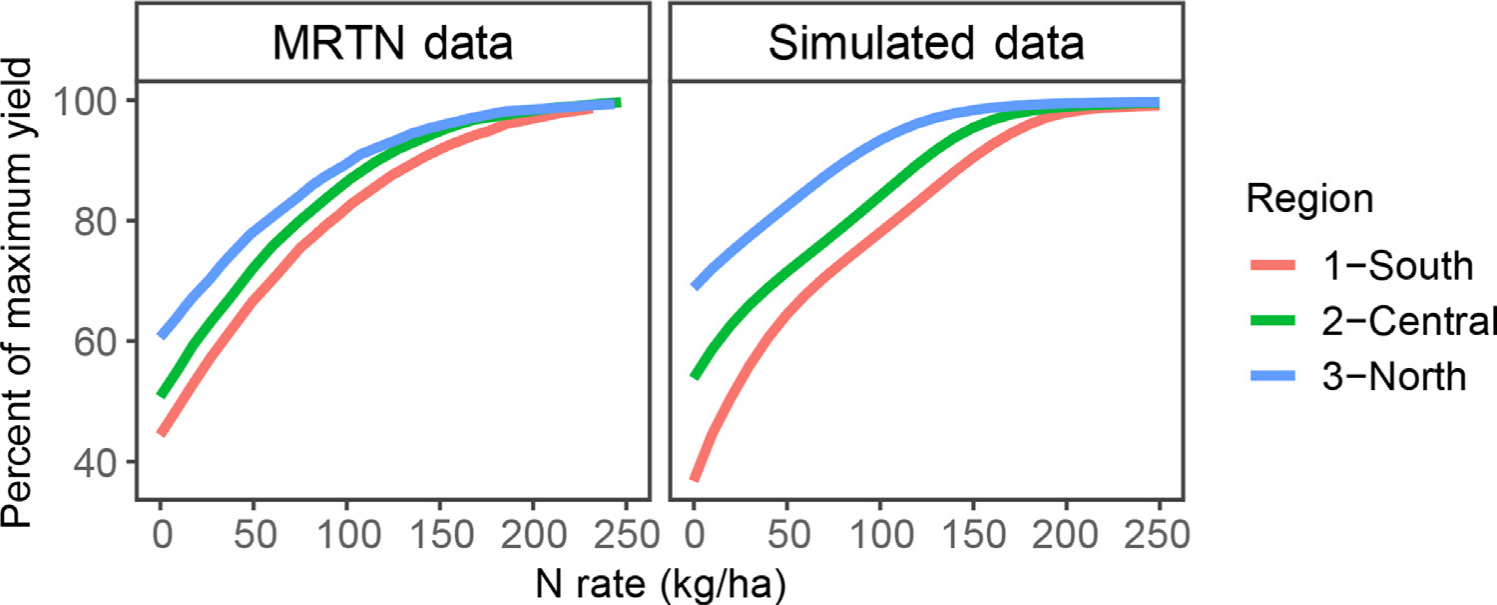
Validation of the average shape by region of the response yield to N, comparing multiple real trials from the MRTN dataset with simulated trials.

**Fig. 3:**
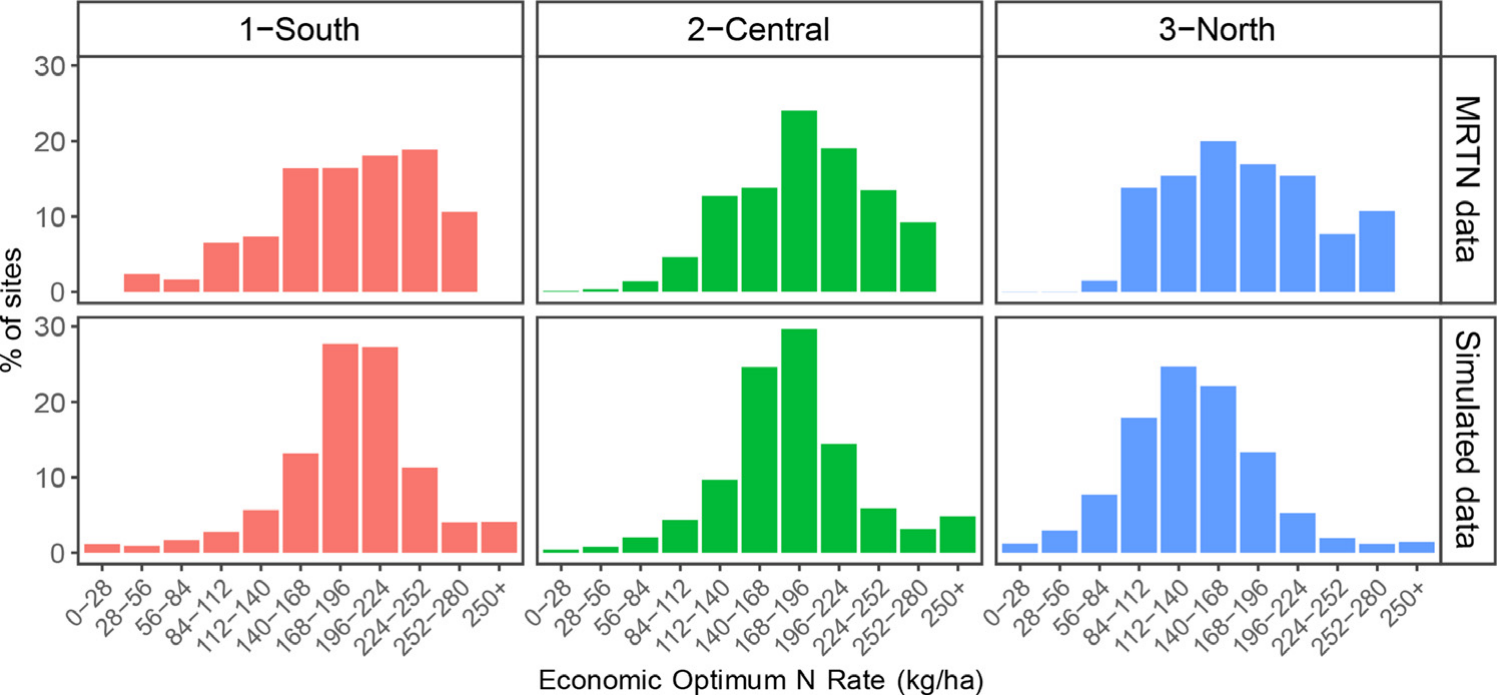
Validation of the EONR distribution by region, comparing multiple real trials from the MRTN data-set with simulated trials.

**Fig. 4:**
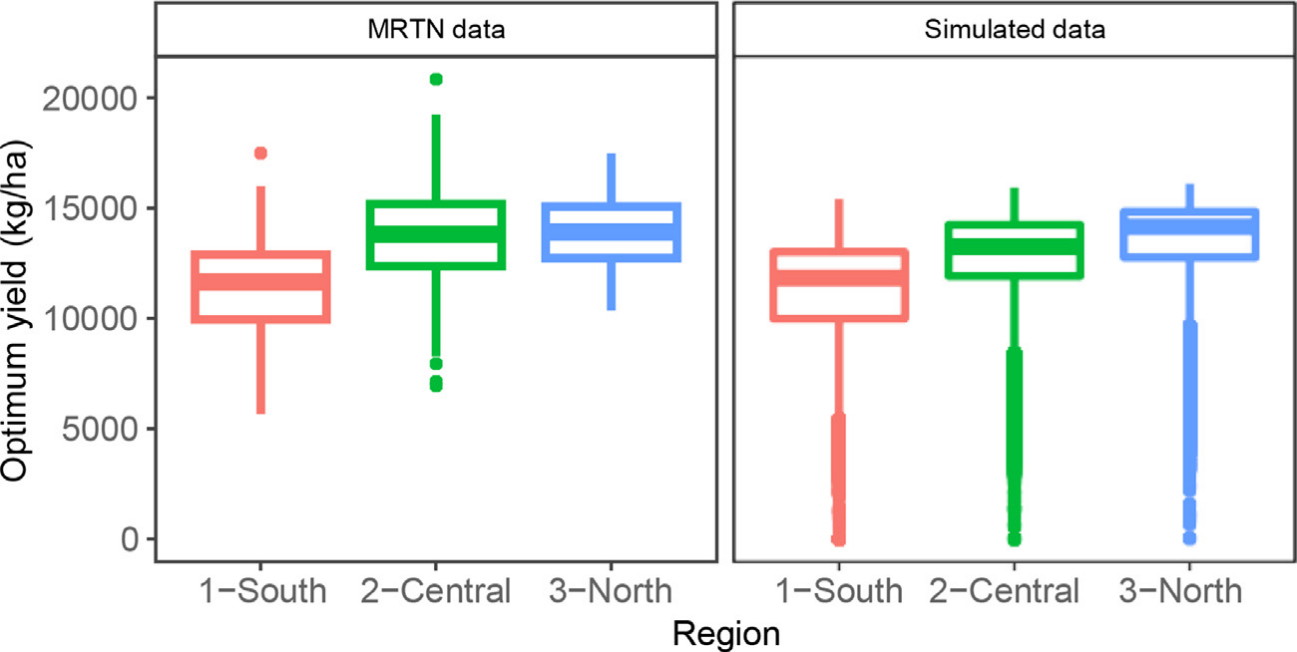
Validation of the yield distribution by region, comparing multiple real trials from the MRTN data-set with simulated trials.

Previous studies have shown that shallow water tables in the US Midwest can have a significant effect on root growth, crop growth, and yield [5]. The simulation of the water table and its impacts on the soil-plant system is complex. Previous field-scale studies used the Richard equation (SWIM model within APSIM) to enable simulation of the water table [5]. However, using this soil water module for big runs across the landscape is challenging because of the lack of physical-based parameters. In this study, we developed a simple approach to account for the impact of the water table using the SoilWat soil water module in APSIM. For this, we included a rule that saturates the soil layer at the water table depth indicated by SSURGO data. The rule was not included in soils that did not have a water table, and in them, a free drainage condition was assumed. If SSURGO informed a water table above 1 meter, it was set at 1 meter because the SSURGO database does not consider installing tile drainage systems in production fields (about 1 m depth) that decrease the depth of the water table to tile depth. This simple addition, increased crop yields in dry years, decreased root depth in wet years, and increased N losses in wet years and overall was a significant addition to more accurate optimal N rate simulation (Fig. 3, Fig. 2, Fig. 4 and 4).

Our approach to simulate a water table allows us to keep the water table at the depth informed by SSURGO. Nevertheless, the SoilWat module does not allow the inclusion of subsurface tile drainage. Tiles had been shown to accelerate N leaching [11], since when the water table reaches them, the water drains from the soil carrying all the dissolved N. To compensate for the absence of tile drainage N losses, we measured N-leaching over the corn that received the fertilizer and the following soybean. This two-year period allowed the excess N to leave the soil.

### 2.3 Management practices configuration

Simulations were conducted for the period 1989–2019, following a corn and soybean rotation, which is the most common cropping system in the Midwest. For that, we numbered the fields on each cell from 1 to 4, and simulations were started so that odd-numbered fields had corn in odd-numbered years and vice versa. This way, approximately half of the fields had corn, and half had soybean each year.

Since our primary goal is to generate the dataset with the response of the crop and environmental variables to increasing N rates, every time a field was assigned to corn, simulations with increasing N rates, from 0 to 320 kg/ha with 10 kg/ha increments were performed -i.e., 33 N rates on each soil every two years. At the end of the period, each field provided fifteen N response curves for each soil it contains, one every two years. All simulations were divided into two-year individual simulations, one for each year x field x soil x N treatment combination. Simulations were started on January 1st on the corn year and extended until December 31st during the soybean year.

Management practices for the crops included tilling of the soil every year on March 20^th^. Corn was planted on the mean historic last frost date of each cell (ranging from April 1st to April 30th). The plant population was nine plants/m^2^. All N fertilizer was applied when the crop reached the stage of five expanded leaves. We used the “B_110” hybrid included in APSIM installation, for which we adjusted the following genetic parameters: radiation use efficiency (rue=2), length from emergence to end of juvenil face (tt_emerg_to_endjuv=185), cycle length from flower to maturity (tt_flower_to_maturity=609), maximum grain number (GNmaxCoef=200), and maximum kernel weight (potKernelWt=300).

For soybeans, the planting date was twenty days after each cell’s mean historic last frost date. This rule also determined the maturity group of the cultivar used. A group III variety (“MG_4”) of seed was planted up to May 5th, after which a group III variety (“MG_3”) was planted up to May 10th and a group II variety (“MG_2”) was planted later than May 10th. The plant population was 30 plants/m^2^, and no fertilizer was applied.

### 2.4 Validation

We used field data from the Maximum Return to Nitrogen (MRTN) [12] calculator tool (available at http://cnrc.agron.iastate.edu/) to calibrate genetic and soil parameters. MRTN is among the most significant trial networks in the area, summarizing multiple N rate trials under different weather years (461 trials at the time of accessing the tool). The MRTN tool divided the state into three regions (southern, central, and northern Illinois) whose soil and crops each differ in their response to N (Fig. 1a), and they show the results of their trials aggregated by region.

We focused on three variables that summarize the response of corn to N. The first one was the shape of the yield response to N, expressed in relative values to the maximum (Fig. 2). The second and third one compared the distribution of the EONR (Fig. 3) and the yield (figure 4) for the base-level condition.

The APSIM model reproduced the response of yield to N in the region accurately, including the year-to-year variations in these variables. Additionally, the simulations captured the differences in south-to-north observed in the state. Two main factors impact this pattern of response. First, the temperature change (a decrease from south to north) delays planting dates and reduces the length of the growing season. Second, the more northern areas have a higher percentage of organic matter in the soil, increasing soil N mineralization. These factors and their interaction are responsible for the lower need for N fertilizer in the northern region, demonstrated by both the higher relative yield with zero N applied (Fig. 2) and the shift of the EONR histogram towards lower N rates (Fig. 3). Simultaneously, deeper soils and milder weather growing conditions create conditions for higher yields (Fig. 4). The yield distribution showed that simulations provided lower values than the observed data. We attribute this to a “trial bias” that could have affected the MRTN experiments, where low-yielding areas were avoided to place a trial, or trials that explored extreme weather were dismissed. We decided to keep the simulations since they are still representative of the growing conditions of the region.

We also validated our simulated state-wide N leaching flow. In this fourth validation, we used a methodology similar to [13] and compared the simulated N leaching for the whole state with averages of N–NO_3_ reported on the Mississippi River near Grafton.

The simulated N leaching consisted of the base-level situation (using N rates recommended by a tool based on the MRTN methodology [1] for the period between 1990 and 2018. The N leaching for the fields was area-weighted averaged for the whole state, considering each cell average area of soybean and corn planted from USDA Crop Frequency Layer.

The streamflow and water nitrate concentration was obtained from the National Water Information System (https://waterdata.usgs.gov/nwis) for the same period (1990–2018). The chosen measurement station is located at Grafton, Illinois (#05587455), and the reason is that this station represents the N loss from the state since it is located at the last point of the Mississippi River in its flow through Illinois. The measurements were cleaned with the following procedure: all values for the same month and year were averaged; if some months did not have values, they were linearly interpolated. Then, the N–NO_3_ concentration was multiplied by water flow to estimate monthly N–NO_3_-flow. Finally, the simulated N-leaching and N-flow monthly values were averaged across the different years into twelve monthly values.

It is important to note that these variables are expected to show a cause-effect relationship since agricultural N loses flow slowly to the Mississippi River. However, there are other sources of N into the Mississippi river other than Illinois cropland, like urban runoff and livestock operations [14]. Additionally, other states located in the Upper Mississippi River Basin also contribute to the streamflow of N at this location. Consequently, we do not expect the relationship to be perfect, but we expect some association between both variables since Illinois agriculture is one of the major sources of N leaching in the mentioned basin.

**Fig. 5:**
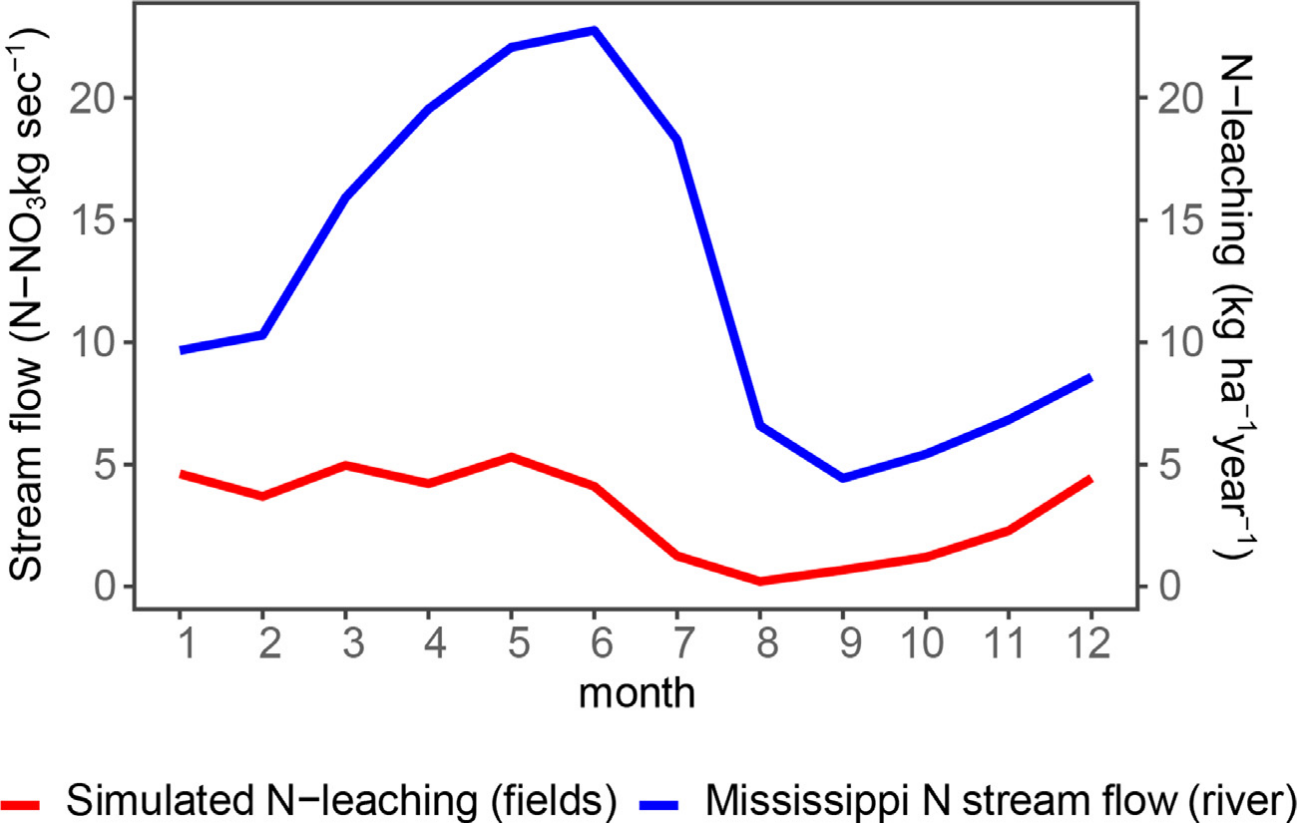
Comparison of simulated N-leaching and reported N–NO_3_ flow at the USGS Gage Station on the Mississippi River near Grafton, Illinois. Monthly flow of both sources was averaged across years.

The time graph shows an association between the simulated N leaching and real N–NO_3_ flow. Moreover, causation is suggested since there is a lag of approximately one month between N leaching peak and valley and the corresponding peak and valley in N-NO_3_ flow (Fig. 5). In early spring, the increase in temperature and rain causes an increase in N mineralization, which, since there is no crop growing at that time of the year, is transported outside of the soil-crop system. At the end of spring, crops start to uptake water and N from the soil, and the flow of N leaching decreases. The flow starts to increase again at the end of the summer when crops reduce uptake when getting closer to maturity. At this time of the year, flow is lower than in spring because temperature decreases, reducing N mineralization and freezing water streams. This validation is encouraging, suggesting that our N leaching simulations capture the pattern of N losses in the state.

## Ethics Statement

This work presented involved extensive crop simulations across the state of Illinois and it did not involve working with animals or humans. This manuscript presents a dataset that is the authors’ original work and co-submitted with the manuscript “Understanding differences between static and dynamic nitrogen fertilizer tools using simulation modeling” published at Agricultural Systems (https://doi.org/10.1016/j.agsy.2021.103275) and is not currently being considered for publication elsewhere. The paper reflects the authors’ own research and analysis in a truthful and complete manner. In addition, the paper properly credits the meaningful contributions of co-authors and co-researchers. All sources used are adequately disclosed. All authors have been personally and actively involved in substantial work leading to the paper and will take public responsibility for its content

## CRediT authorship contribution statement

**German Mandrini:** Conceptualization, Methodology, Software, Validation, Writing – original draft. **Sotirios V. Archontoulis:** Methodology, Validation, Writing – review & editing. **Cameron M. Pittelkow:** Methodology, Validation, Writing – review & editing. **Taro Mieno:** Methodology, Software, Writing – review & editing. **Nicolas F. Martin:** Supervision, Conceptualization, Funding acquisition.

## Declaration of Competing Interest

The authors declare that they have no known competing financial interests or personal relationships which have, or could be perceived to have, influenced the work reported in this article.

## Appendix A Supplementary materials

Supplementary Data S1 Supplementary Raw Research Data. This is open data under the CC BY license http://creativecommons.org/licenses/by/4.0/ Supplementary Data S1
