# Supplementary material for: Simulated dataset of corn response to nitrogen over thousands of fields and multiple years in Illinois
Source: Data Brief. 2021 Dec 28;40:107753. doi: 10.1016/j.dib.2021.107753 (PMC8728579; doi:10.1016/j.dib.2021.107753)
Supplement: Supplementary Data S1 — Supplementary Raw Research Data. This is open data under the CC BY license http://creativecommons.org/licenses/by/4.0/ [file mmc1.pdf]

Agricultural Systems 194 (2021) 103275

Contents lists available at ScienceDirect

Agricultural Systems

journal homepage: www.elsevier.com/locate/agsy

Research Paper

Understanding differences between static and dynamic nitrogen fertilizer tools using simulation modeling

German Mandrini [a], Cameron M. Pittelkow [b], Sotirios V. Archontoulis [c], Taro Mieno [d], Nicolas F. Martin [a,*]

[a] Department of Crop Sciences, University of Illinois at Urbana-Champaign, W201 Turner Hall, 1102 S. Goodwin Avenue, Urbana, IL 61801, USA
[b] Department of Plant Sciences, University of California, Davis, Davis, CA, USA
[c] Department of Agronomy, Iowa State University, Ames, IA, USA
[d] Department of Agricultural Economics, University of Nebraska-Lincoln, Lincoln, NE, USA

HIGHLIGHTS

- Estimating nitrogen rate is critical to balance economic and environmental goals in Illinois corn cropping systems.
- We used a simulated data set to explain the economic and environmental incentives of using complex dynamic tools over simpler static ones.
- We found that complex dynamic tools do not consistently increase profits over simpler static tools.
- Both approaches can reduce N leaching by 15%; dynamic tools by its higher accuracy, static tools by recommending on the low end of the MRTN range.
- Results help re-examine N recommendation goals because aiming for higher accuracy does not necessarily improve profits or reduce N leaching.

GRAPHICAL ABSTRACT

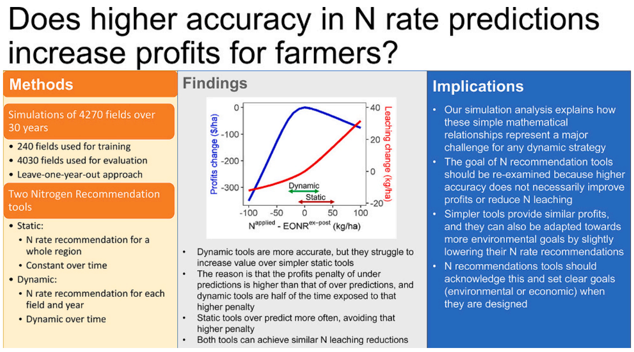

ARTICLE INFO

Edited by Dr Jagadish Timsina

Keywords:
Crop modeling
Machine learning
Environmental indicators
Economic analysis
Maize
Nitrogen fertilizer

ABSTRACT

CONTEXT: Improving nitrogen (N) fertilizer recommendations for maize (*Zea mays* L.) in the US Midwest has been the focus of much research, yet there is no agreement for which methodology is the best to balance trade-offs between production and environmental outcomes. This study investigated the strengths and limitations of two broad approaches: dynamic and static recommendation tools. Dynamic tools use advanced technology to predict the Economically Optimum N Rate (EONR) using year-specific soil, weather, and crop growth characteristics to detect conditions that need lower or higher N rates. Static tools provide regional N recommendations that are static over time, maximizing long-term profits rather than predicting the best EONR for each field and season.

OBJECTIVE: The objective of this work was to explain the interactions between the accuracy, profitability, and environmental losses for different N recommendation tools under a wide range of production scenarios.

* Corresponding author.
E-mail address: nfmartin@illinois.edu (N.F. Martin).

https://doi.org/10.1016/j.agsy.2021.103275
Received 18 April 2021; Received in revised form 12 September 2021; Accepted 14 September 2021
Available online 1 October 2021
0308-521X/© 2021 Published by Elsevier Ltd.

METHODS: For this, we used a calibrated synthetic dataset of 4200 fields over 30 years. In the first part, we compared multiple N recommendations tools belonging to the static and dynamic groups. In the second part, we selected each group's best tools and compared them in detail.
RESULTS AND CONCLUSION: From an economic view, results indicate that increasing profitability by increasing the accuracy in EONR predictions with dynamic tools is challenging. The reason is that these more accurate tools are not perfect, and around half of the time, they under predict. In that situation, the yield penalty is higher, and the economic loss is usually not compensated by savings in N fertilizer costs associated with other more accurate recommendations. The static recommendations avoid this penalty by recommending slightly higher N rates, providing similar profits. From an environmental view, both tools can reduce N leaching by 15%, dynamic tool by being more accurate overall, but the static tool could achieve it by recommending on the lower end of their current recommended N profitable range.
SIGNIFICANCE: Our analysis suggests that we need to re-think the goals of N management tools. Higher complexity in N management may not necessarily increase profits and reduce N leaching. In fact, there is current potential to reduce N leaching by simply reducing static recommendations without hurting profits. For either approach, this study highlights the need to develop other ways (education, environmental awareness, policies) to account for environmental benefits and provide clear incentives for farmers to adopt these tools and increase the eco-efficiency of agriculture.

1. Introduction

Nitrogen (N) is a critical yield-limiting factor in maize (*Zea mays* L.). Great effort has been dedicated to identifying the Economically Optimum N Rate (EONR) under different conditions, which is the rate that produces maximum profits. A key challenge is that the EONR that will maximize profits at harvest (EONR$^{\text{ex-post}}$) depends on the year-specific growing conditions, and it is still unknown early in the season when farmers need to apply N fertilizer to the crop. Therefore, different N recommendation tools (NRT) have been developed to predict it. Decades of research on yield response to N applications have not yet produced an agreement about the best methodology to develop N recommendations for maize in the US (Correndo et al., 2020). Factors causing these inconsistent outcomes include the complexity of the interaction between biophysical processes involved in defining crop yield potential and N demand, the spatial and temporal variability of the main variables driving the response to N addition such as soil N supply, and the uncertainty of the weather that the crop will experience later in the season, long after N applications are made (Tremblay et al., 2012; Morris et al., 2018; Lory and Scharf, 2003; Meisinger, 1984; Zhu et al., 2009). Given this lack of agreement, farmers usually find over-applying N as a solution to guarantee that their crop will not be limited by the nutrient (Vanotti and Bundy, 1994). Overuse of fertilizer N not only decreases their profitability but also increases N losses to the environment, contributing to water quality degradation and a range of other environmental concerns (Vitousek et al., 1997).

Multiple NRTs have been developed to help farmers make better N management decisions. An extensive review of the history, advantages, and disadvantages of several NRT used in the US has been recently published by Morris et al. (2018). Among the tools, there are two big groups to identify EONR. On the one hand, some researchers have developed static recommendations that do not change for particular years or fields and instead are designed to work well on average across a wide range of growing conditions. One example of this approach is the maximum return to N (MRTN) calculator, the current recommendation system promoted in the US Midwest, developed by the extension services from several universities in the area (Sawyer et al., 2006). It is designed to maximize long-term profits to farmers by providing one regional static recommendation, constant across fields and years in a given area (Morris et al., 2018). Another example consists of using the mean EONR from empirical trials in the past to recommend fields in the future. This last approach is not focused on maximizing profits but instead on having the best accuracy possible without considering yearly variability.

The other group of tools uses advanced technology to increase the accuracy of EONR predictions. They are often called dynamic because predictions change in time and space, in contrast to the previous group, which are constant for broad regions and do not change with year-specific conditions. A fundamental assumption of the dynamic approach is that an improved prediction accuracy – i.e., lower prediction error – will save N inputs when possible, improving economic and environmental outcomes. For this, dynamic NRTs use crop, weather, and soil conditions to estimate better the fertilizer needs in each scenario. Dynamic strategies include tools that use soil sampling, canopy sensors, crop modeling and weather information (Ransom et al., 2020; Sela et al., 2017; Puntel et al., 2018; Scharf et al., 2006; Schmidt et al., 2009) to match N inputs with crop demand, improving economic and environmental sustainability. A substantial effort from the public and private sectors is going into these methodologies with some promising results. However, adoption is still low.

Interestingly, the higher complexity of dynamic tools does not seem to always translate into better accuracy, economic and environmental results. Clark et al. (2020) tested the capacity of different soil N tests to predict EONR. It concluded that using soil N information marginally improved maize agronomic predictions and is not suggested as a tool to enhance N fertilizer management in the US Midwest. Qin et al. (2018) found that using current year weather data did not improve predictions compared to using historical weather data, showing the difficulty for recommendation tools to take advantage of precise, in-season information to provide N recommendations. Similar challenges were found by Puntel et al. (2018), who performed predictions using crop modeling software at different stages of crop growth, from planting to maturity. They were successful at predicting yield but obtained an $r^2 = 0.1$ when predicting EONR at the 6th maize leaf stage (v6), concluding that EONR predictions were complex and had greater uncertainty than crop yield prediction. Moreover, in most years, the predictions did not improve later in the season when more weather is known. More recently, Ransom et al. (2020) performed an extensive study covering 49 sites and three years. They found that tools that account for site-specific soil (e.g., PPNT, PSNT) and weather (e.g., Maize-N) were also not better related to EONR than static tools (e.g., MRTN). In this study, MRTN was the tool that maximized profitability. Additionally, dynamic tools did not always show better environmental outcomes, with some dynamic tools showing lower and some higher N losses than MRTN.

All of these results emphasize the limitations of dynamic recommendations and current knowledge gaps. The promise of data-driven agronomy rests on the premise that accounting for soil and weather factors contributing to high variability in crop N response will enable breakthroughs in N management. Research shows that more information and complex prediction tools do not always improve EONR prediction accuracy, economic profits, or environmental outcomes. The reasons are still unclear, and a comparison of tools focusing on identifying where dynamic models perform better and why is not available.

To shed light on this puzzle, studies should be implemented on a

large dataset of soil and climate variability that will result in robust conclusions that are not misguided by particular conditions (Scharf, 2015; Morris et al., 2018). Process-based cropping system models that simulate crop growth in response to soil, water, nutrient, and weather dynamics have been identified as a tool to predict and explain the complex interactions between soil-crop processes (Basche et al., 2016; Sela et al., 2018; Puntel et al., 2018; Lawes et al., 2019). Additionally, these models allow information observed in real trials to be upscaled to larger geospatial domains by calibration and simulation in different environments (Jin et al., 2019, 2018; Baum et al., 2020; Banger et al., 2018), and generate the type of dataset we need to answer the questions that have not been possible to answer using limited trial information.

In this work, we used the Agricultural Production Systems sIMulator (APSIM) (Holzworth et al., 2014), a crop model, to generate and calibrate a database over thousands of fields in Illinois. We trained several NRTs using simulated trials and then evaluated their performance in other fields under different weather conditions. After comparing a set of tools, we focus on investigating the performance of one static and one dynamic tool for (i) explaining the trade-offs between profits, accuracy, and N losses and (ii) dissecting how dynamic models create value over static models, recognizing conditions where the model is more likely to provide positive value. We hypothesize that advanced models with higher accuracy, dynamic NRTs, do not increase profits over more straightforward long-term profit-maximizing tools, static NRTs. We believe that our findings apply to the comparison of static and dynamic tools in general, allowing researchers to find opportunities to improve the eco-efficiency of NRTs by reducing environmental N losses without decreasing farmers' profits. Our findings will also be significant for policymakers to create incentives for developing and adopting models that can reduce N leaching without hurting farmers' profitability.

2. Materials and methods

2.1. Dataset

For this work, we used the dataset published and publicly available in Mandrini et al. (2021). The database consisted of simulations for 4270 fields located in the state of Illinois. Historical weather was obtained for the period 1989–2019 (30 years long) from DAYMET (Thornton et al., 2014). Soil information for the three dominant soils on each field was obtained from the USDA Soil Survey Geographic Database (SSURGO) (Natural Resources Conservation Service, 2018). The fields had a maize-soy rotation, with approximately half of them having maize in odd-numbered years and soybean on even-numbered years, and vice-versa for the other half. On each field, each time it was assigned to maize, the response to increasing N rates, from 0 to 320 kg/ha with 10 kg/ha increments, was simulated (33 N rates in total) using APSIM. All N fertilizer was applied when the crop reached the stage of five expanded leaves (stage of v5). Simulations were run for each of the three most dominant soils on each field. Finally, all the variables were aggregated at the field level, doing an area-weighted average using the area of each soil as weighting variable, obtaining the final database characterized in Table 1.

The database was calibrated and validated using more than 400 trials performed in the region over multiple years. The validation showed that the yield response to N provided by APSIM are representative of the responses obtained in the different soils and weather-years of the state and that the simulated N-leaching represents state-wide N leaching patterns observed by flow measurements in the Mississippi River.

We calculated profits using the following equation:

$$\text{Profits} = \text{Yield(kg/ha)}\ \text{Pc(\$/kg)} - N\text{(kg/ha)}\ \text{Pn(\$/kg)} \tag{1}$$

where *Yield* is the yield of maize (kg/ha). *N* is the N rate (kg/ha). *Pc* is the price of maize, set to 0.158 $/kg grain (equivalent to 4.00 $/bu). *Pn* is the price of N fertilizer set to 0.88 $/kg N (equivalent to 0.40 $/lbs N).

Table 1
Database characterization. Each row represented a simulation for one field in one year with one N rate, with a total of 4,227,300 rows (4270 field × 30 years × 33 N rates).

Variable	Description	Units	Use	Group
Region	Region identification (1-South, 2-Cental, 3-North)	–		
id_10	Cell identification number	–		
id_field	Field identification number (1–4)	–		
Station	Trial field (1) or evaluation field (0)	–		
Year	Year of the maize simulation (1989–2018)	–		
N_fert	Nitrogen added as fertilizer in v5	kg/ha	Treatment	
Yield	Yield of the maize in with 15% Moisture	kg/ha	Response	
L	Total 2-years N leaching during maize and soybean. From April 1st year (x) to March 31st year (x+2)	N kg/ha	Response	
day_sow	Planting date	Julian date	Predictor	Low
day_v5	Date when the maize reached v5	Julian date	Predictor	Low
lai_v5	Leaf Area Index at v5	m^2/m^2	Predictor	Low
rad_1	Average solar radiation during first period (1 Jan. to planting)	$MJ/m^2/day$	Predictor	Low
rad_2	Average solar radiation during second period (planting to v5)	$MJ/m^2/day$	Predictor	Low
rain_1	Total precipitation during first period (1 Jan. to planting)	mm	Predictor	Low
rain_2	Total precipitation during second period (planting to v5)	mm	Predictor	Low
surfaceom_wt_v5	Surface residue weight at v5	kg/ha	Predictor	Low
tmean_1	Average air temperature during first period (1 Jan. to planting)	°C	Predictor	Low
tmean_2	Average air temperature during second period (planting to v5)	°C	Predictor	Low
Y_maize_lt_avg	Mean yield at EONR (for the other 29 years)	kg/ha	Predictor	Low
clay_40cm	Clay content (0-20 cm)	%	Predictor	High
n_0_60cm_v5	Soil N (NO_3 and NH_4) from 0 to 60 cm at v5	kg/ha	Predictor	High
oc_20cm_v5	Soil Organic Carbon at v5 (0-20 cm)	%	Predictor	High
sand_40cm	Sand content (0-20 cm)	%	Predictor	High
sw_dep_v5	Soil water content at v5	mm	Predictor	High
whc	Water holding capacity	mm	Predictor	High
rad_3	Average solar radiation during third period (v5-R1)	$MJ/m^2/day$	Predictor	Future
rad_4	Average solar radiation during fourth period (R1-R3)	$MJ/m^2/day$	Predictor	Future
rad_5	Average solar radiation during fifth period (R3-R6)	$MJ/m^2/day$	Predictor	Future
rain_3	Total precipitation during third period (v5-R1)	mm	Predictor	Future
rain_4	Total precipitation during fourth period (R1-R3)	mm	Predictor	Future
rain_5	Total precipitation during fifth period (R3-R6)	mm	Predictor	Future
tmean_3	Average air temperature during third period (v5-R1)	°C	Predictor	Future
tmean_4	Average air temperature during fourth period (R1-R3)	°C	Predictor	Future
tmean_5	Average air temperature during fifth period (R3-R6)	°C	Predictor	Future

These prices are standard assumptions and have been used previously (Ransom et al., 2020). For simplification, we did not account for other costs related to N management -i.e., cost of application, cost of obtaining soil and weather variables used as predictors, cost of implementing field trials.

2.2. General flowchart

This paper combined simulated data and economic analysis to evaluate decisions about optimal N management practices. Similar frameworks have proven to be useful for this type of research (Finger, 2012; Kuhn et al., 2010; Semaan et al., 2007; McNunn et al., 2019).

Our training and testing process was a combination of leave-one-year-out together with a split of the fields into 240 trial fields and 4030 evaluation fields (Fig. 1a). Each year in the 30-year-long sequence, three stages were followed in a hypothetical progression of developing N rate recommendations: (1) The information from trial fields of crop response to multiple N rates for all the years except the one evaluated was filtered from the database, (2) these responses were used to calculate economic returns and develop several NRTs that predict the optimal N rates, and (3) these optimal N rate recommendations were applied to the evaluation fields during the year that was left out from stage 1 to evaluate the performance of these tools. With this last step, the APSIM output for the recommended N rate was filtered from the database. In summary, the trial fields were always the same, and the optimal N recommendations generated by different NRTs were evaluated in other fields called evaluation fields and in different weather-years by the leave-one-year-out approach.

2.3. Stage 1: trial data

For each year, the data from the trial fields for all the years except the one left out was filtered. The data consist of 240 trial fields of maize response to multiple N rates, from 0 to 320 kg/ha, with 10 kg/ha increments. Because the trial fields also followed a maize-soy rotation, half of them provided N response curves on odd-numbered years and the other half on even-numbered years. In total, the filtered trial data consisted of 3480 N response curves, covering 29 weather-years.

2.4. Stage 2: optimization module

We used the trial data from the previous stage to optimize static and dynamic recommendation tools.

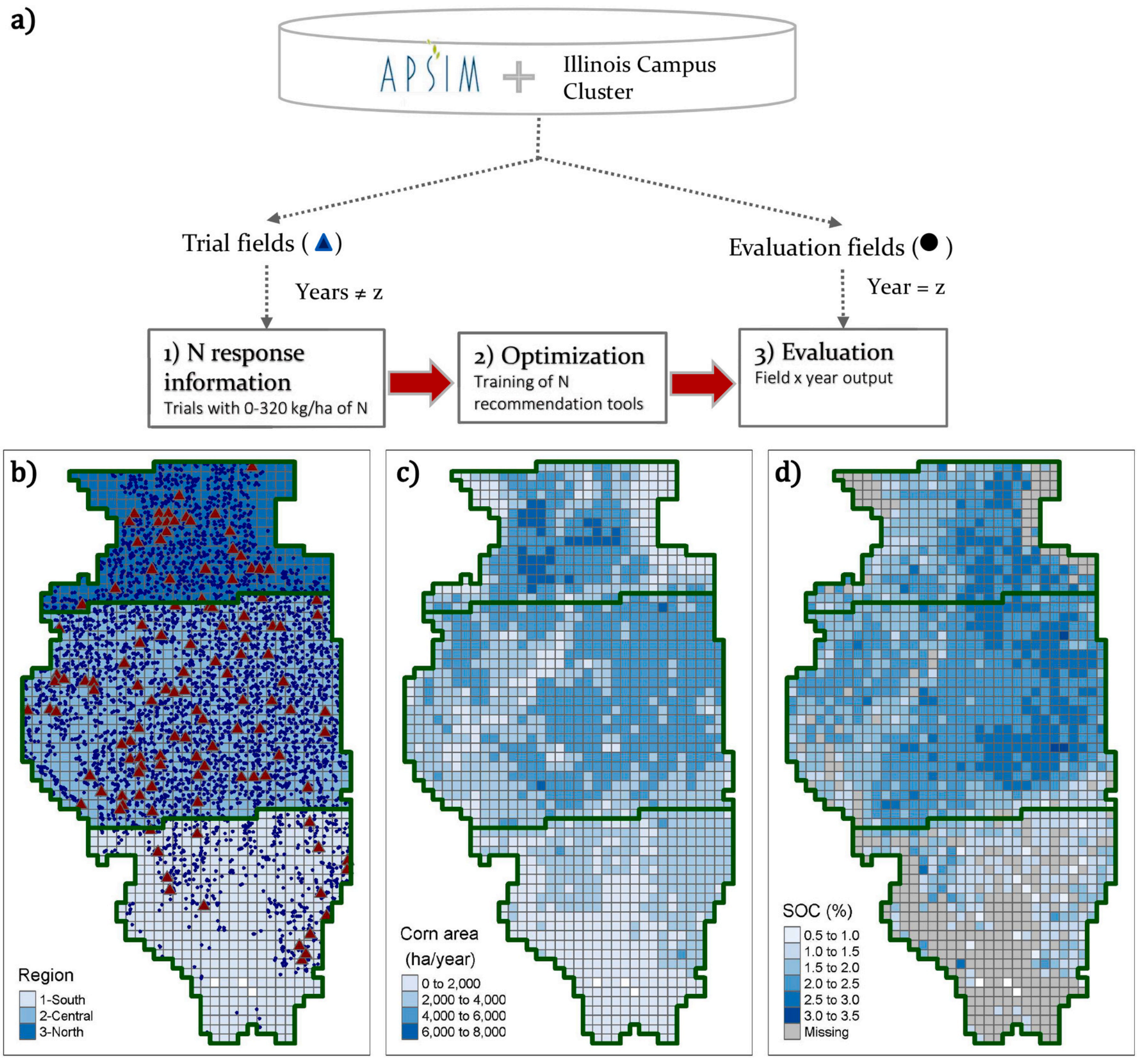

Fig. 1. (a) Schematic diagram of the flow chart followed in the analysis. Boxes indicate the major processes. Arrows indicate the flow of information. (b) Map of Illinois, showing the grid of 10 × 10 km cells, the three regions, the trial fields (big red triangles), and the evaluation fields (small blue dots). (c) Map of Illinois showing the average area planted to corn according to the USDA Crop Frequency Layer (Boryan et al., 2011). (d) Map of Illinois showing the average soil organic carbon 0–20 cm for the soils used in the simulations.

2.4.1. Static recommendations

The MRTN calculator is a recommendation tool introduced in 2005 by the extension services of seven Universities in the US Corn Belt (Sawyer et al., 2006; Morris et al., 2018). The recommendations are available online, but instead of directly using the MRTN output, we applied the MRTN methodology to our data. The reason is that we wanted to evaluate this methodology against others, performing a fair comparison and avoid confounding effects that would arise by evaluating recommendations obtained from real data against those using simulated data.

Therefore, we followed the steps described in Sawyer et al. (2006) to calculate MRTN recommendations with our dataset. Initially, the state of Illinois was divided into three regions, identified to have similar crop growing environments (Fig. 1). In each region, we used the trial data to calculate the average profits for each N rate (that is, for each N rate, the profits across all the trials and years were averaged). Then, the N rate that maximized those averaged profits was identified and used in the region (NRT = mrtn_peak). Additionally, the MRTN methodology recognizes the existence of a flat payoff function where the return to N is almost constant. Therefore, the prediction of an exact optimal N rate has inherent uncertainty, and the MRTN provides a profitable range within 2.47 $/ha (1.00 $/acre) from the peak. We calculated both ends of the range and implemented a recommendation tool using the low end (NRT = mrtn_low) and another at the high end (NRT = mrtn_high). After conducting a state-wide survey, Sellars et al. (2020) reported that 67% of fields in Illinois received N fertilizer rates above mrtn_peak recommendations. Moreover, other researchers have assumed that N rates actually applied by farmers are closer to the high end of the profitable MRTN range (Millar et al., 2010; Basso et al., 2019).

One adaptation in our work is that the original MRTN methodology fits a quadratic-plateau model to the data to interpolate yield between different N rates tested in the trials. We did not need to interpolate because our trials had a 10 kg/ha step between rates, and that precision was enough for our evaluation. This approach minimizes model fit errors that are known to be substantial. A similar approach was followed in previous works (Puntel et al., 2016, 2018).

We included an even simpler static methodology that uses the mean EONR from the trial data in each region (NRT = eonr_mean). This methodology has a two-step algorithm. First, the EONR for each trial year was obtained. Second, all the EONRs for the same region were averaged, and that is the final recommendation. In theory, this tool should have higher accuracy than MRTN, since it selects the mean EONR, instead of the N rate that maximizes long-term profits.

2.4.2. Dynamic recommendations

The dynamic strategies were new N management strategies we developed to predict better EONR by incorporating soil and crop year-specific conditions into the predictions. They are based on a machine learning technique called "Random Forest" (Breiman, 2001). Random Forest is a learning method for classification and regression that operates by building a regression tree by sampling the training data and splitting it into many subgroups called nodes. The tree is grown so that, at each of these splits, the data is divided by finding the variable and threshold that increases the purity of the child nodes. Many trees are built with different samples of observations and different variables. Then, the average of all the trees is the prediction of the model. This methodology is efficient at capturing non-linear relationships in the data, like those encountered in crop responses to weather, soil, and N rates. Additionally, since many trees are built using different samples and variables, it is robust to overfitting. Recent work has demonstrated the capacity of machine learning models to predict maize N demand (Qin et al., 2018; Lawes et al., 2019), the yield of maize without N fertilization (Correndo et al., 2020) and crop yields and N losses using a similar approach (Shahhosseini et al., 2019). The Random Forest models were built using the package RandomForest in R (Liaw and Wiener, 2002).

For each of the 3480 trials in the trial data, the N rate that maximized profits ($EONR^{ex\text{-}post}$) was selected. This filtered data was the training dataset, where each row was a trial, and the columns contained several predictor variables and the $EONR^{ex\text{-}post}$ as the response variable. The predictor variables were classified into three sets (Table 1). The low set describes soil and weather conditions that farmers can easily obtain without significant cost. The high set includes variables that require more time and effort to be obtained. Finally, the forecast set includes the precise future weather information influencing crop growth and potential N losses (temperature, rainfall, and radiation) for the field. The first two sets include only information before the 5th maize leaf stage (v5) as it reflects reality better (the summer weather is unknown when farmers and agronomists make decisions). The forecast set includes future weather information beyond the possibilities of current forecasts and was included to test how having such information would improve predictions.

Three Random Forest tools were trained using different sets of predictor variables. The first uses the low information set of variables (NRT = rf_reduced). The second involves the previous variables plus the high information set (NRT = rf_full). The third includes the two previous sets of variables plus the forecast set (NRT = rf_future).

The importance of the different predictor variables for the random forest was measured using the varImpPlot function in the R "randomForest" package. The varImpPlot function calculates the decrease in node impurities from splitting on the variable, averaged over all trees. For regression, the node impurity is measured by the residual sum of squares. Important variables are identified as those that reduce the residual sum of squares in the splits on which they were used (the difference between residual sum of squares before and after the split). More details about the hyperparameters, together with a variable importance plot is provided in Supplementary Materials (Sections S1.1 and S2.1).

The last dynamic tool uses the exact $EONR^{ex\text{-}post}$, which is the N rate that maximized profits – i.e., at harvest – in that field and year (NRT = ex_post). Currently, this is not feasible, but this tool was included as a benchmark for comparison. In theory, this tool would show the best possible outcome if we could predict the N rate under full information.

NRTs reference:

- Static tools:
  1. $eonr\_mean$ = mean of past EONRs from trials.
  2. $mrtn\_low$ = low end range of MRTN methodology.
  3. $mrtn\_peak$ = most profitable rate of MRTN methodology.
  4. $mrtn\_high$ = high end range of MRTN methodology.
- Dynamic tools:
  5. $rf\_reduced$ = random forest with low information variables.
  6. rf_full = random forest with low, and high information variables.
  7. rf_future = random forest with low, high and forecast information variables.
  8. ex_post = EONR under full information (i.e., $EONR^{ex-post}$ for each field and year).

2.5. Stage 3: evaluation module

Different metrics were used to evaluate the performance of each tool for providing N rate recommendations. Accuracy is the capacity of a tool to make predictions that are close to the $EONR^{ex\text{-}post}$. It was measured using the mean error (ME), the mean absolute error (MAE), and the root mean squared error (RMSE) (see equations in the Appendix S1.2). The mean error shows the directionality of the error – i.e., on average, does the tool over or under predict. The MAE shows the magnitude of the absolute errors without considering directionality. The same applies to the RMSE, but it emphasizes extreme errors rather than those close to the observed value by squaring them.

The environmental performance was evaluated by the potential reduction in N leaching, which was the total leaching during the two

years period, including the maize year when the N was applied and the following soybean year, to capture any residual effect of the N rate (Iqbal et al., 2018; Pasley et al., 2021; Mandrini et al., 2021). The N leaching was shown as an absolute value (kg/ha) and a relative value (%) relative to the base-level situation. This study assumed that the base-level situation was the N leaching obtained from the mrtn_high (Millar et al., 2010; Basso et al., 2019; Sellars et al., 2020). Finally, the economic performance was evaluated by the partial profits achieved with the different NRT (Eq. (1)).

To estimate statistical differences between NRTs, we performed a hierarchical mixed models analysis with package lme4 from R (Bates et al., 2015). We considered as a fixed factor the NRTs and as random factors the weather-year, the cell, and the field. The model accounts for the year effect, the cell nested inside the year (because the year will have a significant effect – i.e., 2012 was dry for all cells – but there is spatial variation in the weather between the cells), and the field nested inside the cell (the four fields in a cell had the same weather, but different soils). The fields by year were the unit of observations. We determined significant differences by Tukey-adjusted comparisons (p-value = 0.01).

In the second part of the work, we focus on comparing the performance of the best ex-ante dynamic tool (rf_full) and the best static tool (mrtn_peak). We calculated the value of dynamic recommendations as the difference in the profits using the following equation:

$$\text{Dynamic value} = \text{Profits}_{cfz}^{rf\_full} - \text{Profits}_{cfz}^{mrtn\_peak} \quad (2)$$

where $\text{Profits}_{cfz}^{rf\_full}$ are the profits for the cth cell, the fth field, and the zth weather-year using the N rate recommended by the rf_full tool. $\text{Profits}_{cfz}^{static-mrtn}$ are the profits for the cth cell, the fth field, and the zth weather-year using the N rate recommended by the mrtn_peak tool.

3. Results

3.1. N recommendation tools comparison at the state level

The $\text{EONR}^{\text{ex-post}}$ has considerable variability between different years, with years for which the median N rate was 120 kg/ha and others it was 220 kg/ha (Fig. 2). Moreover, for a given year, the variability of rates between different fields can be small (as in 1998) or very large (as in 1996). This figure shows the difficulties of predicting the EONR, since there is significant variation, driven mainly by unpredictable weather and unknown soil conditions early in the season.

The aggregated results over 4030 fields and 30 years show that the rf_full was the most profitable tool among the tools trained with information available to farmers when making the N rate decision, which we considered to be the crop stage of v5 (Table 2). If knowing the full season weather (NRT = rf_future) was possible, it would increase profits by 4 $/ha and reduce N leaching by 0.1 kg/ha. Knowing the precise $\text{EONR}^{\text{ex-post}}$ (NRT = ex_post) would increase profits by another 20 $/ha, and reduce N leaching by an extra 1.4 kg/ha. These last two tools are outside real possibilities and were included to measure the potential benefit of perfect forecasts in the first case, and by perfect N management in the second case. The ex_post tool sets the maximum profits that a perfect prediction of N rates can obtain.

The most profitable static tool was the mrtn_peak, closely followed by eonr_mean, the mrtn_high, and the mrtn_low. Surprisingly, the rf_reduced, which uses low information variables as predictors, had lower profits than the static methods. This suggests that if dynamic recommendations are going to be used, they need to be based on better information that requires more effort and cost (i.e., soil sampling).

Interestingly, tools that achieved similar profits have large variations in N leaching outcomes (Table 2). For example, the mrtn_high and mrtn_low had the same profits, but N leaching decreased from 44.1 kg/ha to 37.4 kg/ha (15.2%). The important aspect to observe is that this reduction could be obtained at no cost.

3.2. Dynamic versus static N recommendations comparisons

When farmers adopt a complex N recommendation tool that requires collecting information, the general expectation is that those recommendations will provide value (as defined in (Eq. (2)). The complex rf_full increased the accuracy of the predictions (lower RMSE), but it narrowly provided 2 $/ha of value over the simpler mrtn_peak across all the fields and years (Table 2).

These two tools represent the two broad approaches to N rate recommendations described in the introduction. In this second part of the work, we compared them to understand why the dynamic tool did not increase profits over the simpler static tool. The reason for selecting the rf_full tool is that it was the most profitable of the dynamic group with the information available to farmers when making the decision, whereas rf_future and ex_post use information that is not realistically available. The reason for selecting the mrtn_peak is that it was the most profitable static tool.

3.2.1. Diagram of average profits and leaching response

One crucial characteristic of N management is that under-applying N is more harmful to profits than over-applying. Fig. 3a shows a response of profits and leaching to the N residual -i.e., the difference between the

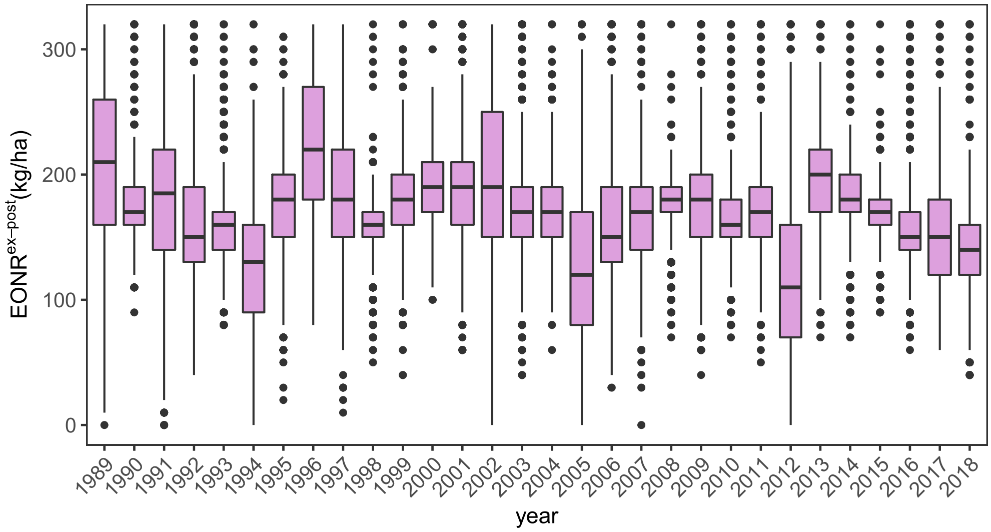

Fig. 2. Simulated $\text{EONR}^{\text{ex-post}}$ distribution by year. The same cultivars were used across years. The lower and upper hinges correspond to the first and third quartiles (the 25th and 75th percentiles). The middle line shows the median. The upper and lower whisker extends from the hinge to the value no further than 1.5 * IQR from the hinge (where IQR is the interquartile range, or distance between the first and third quartiles). Data beyond the end of the whiskers are called "outliers" points and are plotted individually. *Source*: 4030 evaluation fields by 30 weather-years.

Table 2
State averages of Yield, N leaching, N rate and Profits. Different letters show significant differences for each variable for the mixed models ($p - value < 0.01$). Leaching change shows the leaching relative to the base-level situation (mrtn_high). Overpred, subpred are the proportion of fields by year combinations that the model predicted above and below the $EONR^{ex-post}$.

NRT	Yield (kg/ha)		N Leaching (kg/ha)		(% change)	Profits ($/ha)		N rate (kg/ha)		N rate min (kg/ha)	N rate max (kg/ha)	ME (kg/ha)	MAE (kg/ha)	RMSE (kg/ha)	r^2	Over pred (%)	Sub pred (%)
ex_post	12703	a	36.3	g	−17.7	1857	a	171	e	0	320	0	0	0	1.00	0	0
rf_future	12588	c	37.7	de	−14.5	1837	b	172	d	60	260	2	29	41	0.35	48	38
rf_full	12559	d	37.8	d	−14.3	1833	c	172	d	50	250	2	33	46	0.17	50	39
mrtn_peak	12588	c	39.9	b	−9.5	1831	d	180	b	160	210	9	36	50	0.07	56	34
eonr_mean	12542	e	37.6	e	−14.7	1830	e	172	d	140	200	1	34	48	0.11	48	40
mrtn_high	12664	b	44.1	a	0.0	1829	f	195	a	170	220	25	42	54	0.10	72	20
mrtn_low	12521	f	37.4	f	−15.2	1829	f	170	f	140	200	−1	35	49	0.08	45	44
rf_reduced	12518	f	38.3	c	−13.2	1825	g	173	c	60	260	3	37	49	0.08	50	40

NRTs reference: Static tools: *eonr_mean* = mean of past EONRs from trials. *mrtn_low* = low end range of MRTN methodology. *mrtn_peak* = most profitable rate of MRTN methodology. *mrtn_high* = high end range of MRTN methodology. Dynamic tools: *rf_reduced* = random forest with low information variables. *rf_full* = random forest with low, and high information variables. *rf_future* = random forest with low, high and forecast information variables. *ex_post* = EONR under full information (i.e., $EONR^{ex-post}$ for each field and year).

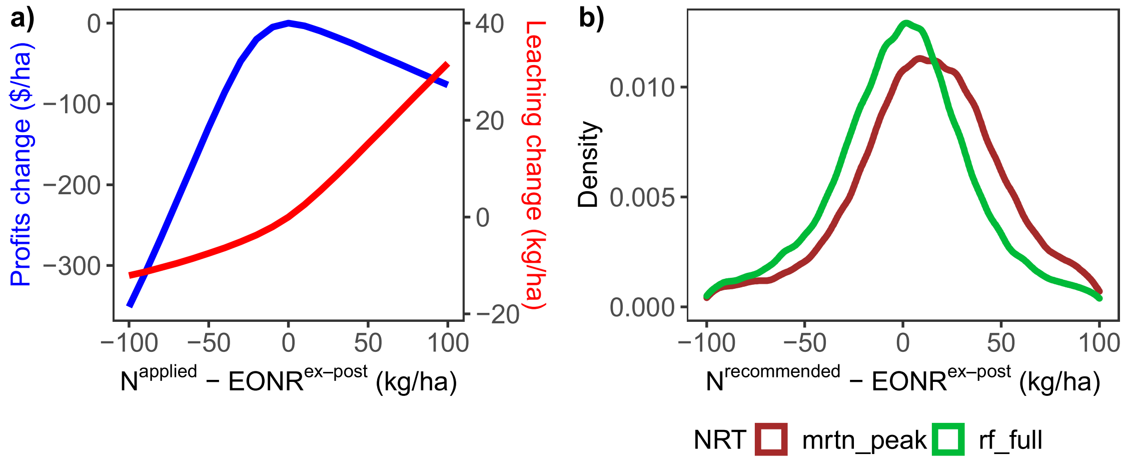

Fig. 3. (a) Response of profits and leaching to the difference between the N applied in a trial and the $EONR^{ex-post}$. Profits change = Profits$N^{applied}$ - Profits$EONR^{ex-post}$. Leaching change = Leaching$N^{applied}$ - Leaching$EONR^{ex-post}$. *Source*: 240 trial fields by 30 weather-years. (b) Density plot of the difference between the N rate recommendation for a field and the $EONR^{ex-post}$ for two N recommendation tools. Density plots show the distribution of a continuous variable, standardized so the sum of the area under the curve equals 1. *Source*: 4030 evaluation fields by 30 weather-years with leave-one-year-out approach.

N rate applied in a trial and the $EONR^{ex-post}$. The profits are the maximum at the EONR, when the residual is zero. To the left, profits are lower due to a yield penalty for not having enough N. To the right, profits are lower due to a cost penalty for adding N fertilizer that did not increase yield. The characteristic to notice is that the decrease to the left is greater than to the right of the EONR since the yield penalty is higher than the cost savings associated with a reduction in N rate. On the other hand, the leaching curve path is different, and it always increases when the N rate is higher, and it increases linearly after the EONR, since the crop is close to maximum uptake capacity and the extra N is lost from the soil-crop system.

Current mrtn_peak has an RMSE of 50 kg/ha, over predicting 56% of the time (Table 2). The more eco-efficient rf_full has an RMSE of 46 kg/ha, over predicting 50% of the time. Nevertheless, both strategies had similar profits. Part of the reason can be found in the shape of the profits curve. The mrtn_peak is less accurate and tends to over predict, but since the loss in profits to the right is lower than to the left, it provides the same profits when aggregated across all fields. The rf_full recommends with higher accuracy, but half of the recommendations are below the EONR, which has a larger impact on profits, overcoming the economic gains due to higher accuracy. However, the N leaching for the rf_full is 5% lower than the current mrtn_peak. The reason is that the average N rate recommended by the rf_full was lower than the average N rate recommended by the mrtn_peak.

When we compare NRTs that recommend N rates close to the EONR, slight differences in methodologies can lead to different outcomes, prioritizing profitability or environmental impact, but not both. Fig. 3 provides two reasons for this characteristic. First, better accuracy does not increase profits, and models biased to the right of the EONR can have similar profits than more accurate models that recommend half of the time above and half of the time below the EONR. Second, better accuracy does not necessarily mean lower N leaching either, since leaching depends on the directionality of error. That is, leaching is higher with predictions above EONR (positive errors) but lower with predictions below EONR (negative errors). The main consequence of these two relationships is that choosing N recommendations tools that maximize profits does not necessarily reduce N leaching. Therefore, N management experts and farmers need to have clear goals (profits vs. environmental) and indicators (i.e., accuracy, profits, or reduction in N losses) when comparing N recommendation tools.

Another consequence of the explained trade-offs is the low ability of MAE and RMSE to compare N recommendation tools that are already close to the optimal N rate. These indicators use absolute errors and, therefore, do not capture their directionality. Since directionality is important, models with similar MAE or RMSE can be biased towards economic outcomes (if the errors are positive) or environmental

outcomes (if the errors are negative). Consequently, we recommend complementing these commonly used measures of model performance with other indicators that show directionality, like the ME, to provide more valuable comparisons of models.

3.2.2. Dynamic value disaggregation into dynamic value per outcome and frequency of those outcomes

This section looks at the dynamic value in more detail, examining its magnitude when rf_full recommendations fail or succeed with respect to the static mrnt_peak. We also assess the N rates that provided those outcomes and how often they happened. For this, we expressed the N rates as N rate difference (N^{rf_full} - N^{mrnt_peak}). We divided the N rate difference into five bins, going from -60 to 60, with 30 kg/ha increments. There are two bins below mrnt_peak, two above mrnt_peak, and another bin for the zero difference (i.e., when both tools recommended the same N rate). Note that the N rate difference is a separate variable from the previous N residual, where N rates were subtracted from the $EONR^{ex\text{-}post}$ and not compared directly to the mrnt_peak.

The dynamic value, calculated with Eq. (2), was disaggregated into two components. The multiplication of them is the final value per ha of dynamic recommendations. The first component is the dynamic value per outcome (Fig. 4a). It is the value of Eq. (2) split into categories based on whether the prediction was a success or failure compared to the static recommendation (outcome) and whether the N difference was positive or negative, small or large (N rate difference bin). This allows comparing the magnitude of the dynamic value gain when the rf_full successfully outperformed mrtn_peak (positive dynamic value), with the magnitude of the dynamic value loss when rf_full failed to outperform mrtn_peak (negative dynamic value).

Results show that, for negative N rate differences, the dynamic value loss by a failed recommendation is larger than the dynamic value gain by a successful recommendation. On the other hand, the pattern reverses for positive N rate differences, and the dynamic value lost by a failed recommendation is smaller than the dynamic value gained by a successful recommendation for negative N rate differences. This change in the dynamic value per outcome from negative to positive N rate differences is explained by the previous average diagram (Fig. 3a). When the rf_full recommends and fails on rates below the mrtn_peak, there is a higher chance of also being below the $EONR^{ex\text{-}post}$, thus experiencing a higher loss than when failing above the mrtn_peak.

The second component is the frequency of each outcome: success, failure, or neutral (Fig. 4b). This is the proportion of field-by-year combinations that the rf_full recommendation falls in each group (N rate difference by outcome). Interestingly, most of the successful rf_full recommendations occur on the negative N rate differences, which is explained by the fact that mrtn_peak recommendations are designed to maximize profits in the long term, and for that, they recommend rates that are slightly above the mean N rate (180 kg/ha is the mean mrtn_peak recommendation, while 172 kg/ha is the eonr_mean, Table 2).

The rf_full tool had a higher proportion of successes on the negative N differences, but since the dynamic value loss (i.e., economic penalty) by the failures is larger than the gain of the successful ones (N rate difference < 0 in Fig. 4a), the final dynamic value decreased (Fig. 4c). Depending on the frequency of success vs. failure, the positive N rate differences could compensate for that, given the more beneficial

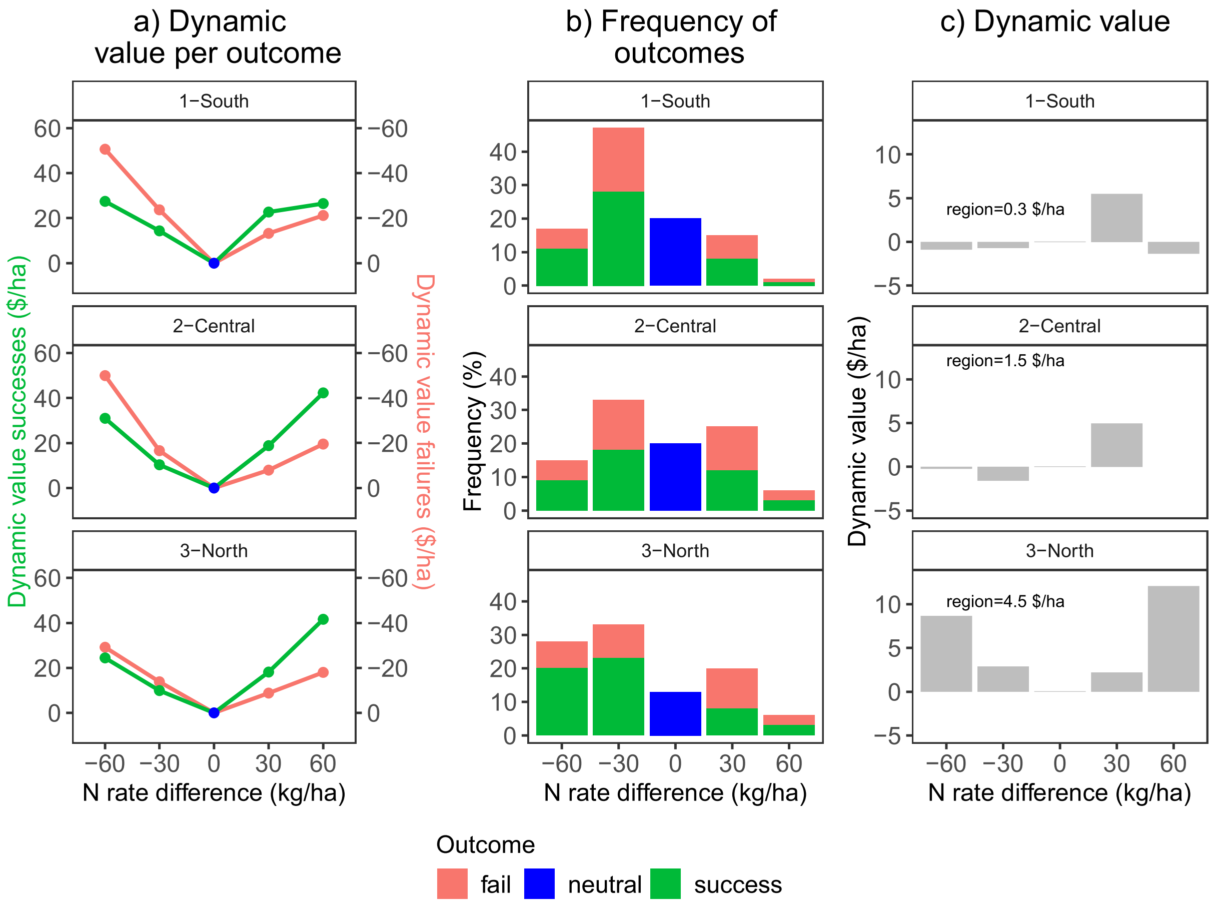

Fig. 4. Components of the dynamic value. N rate differences ($N^{rf\_full} - N^{mrtn\_peak}$) were grouped in bins (−60 to −30, −30 to −1, 0, 1 to 30, 30 to 60 kg/ha). (a) Dynamic value per outcome (dynamic value of Eq. (2) calculated by outcome, N bin and region). (b) Frequency of outcomes. (c) Dynamic value (mean dynamic value for all fields in a N bin in a region). The text shows the mean dynamic value for the region (mean dynamic value for all fields in the region). *Source*: 4030 evaluation fields by 30 years with leave-one-year-out approach.

dynamic value per outcome (N rate difference > 0 in Fig. 4a). Nevertheless, dynamic predictions occur less often on the positive side and have a lower frequency of success, resulting in an overall economic loss for negative N differences.

When comparing by region, dynamic recommendations provided higher value when moving north (south = 0.3 $/ha, central = 1.5 $/ha, north = 4.5 $/ha; Fig. S1). Two reasons help explain this effect. The first reason is the higher accuracy of the rf_full tool. This can be seen by a marginally better RMSE when moving north (south = 50 kg/ha, central = 47 kg/ha, north = 42 kg/ha; Table S1). To understand why the model is more accurate when moving north, we trained individual rf_full for each region and compared the variance explained by the model, which is an output from the "randomForest" package in R. The percentage of variance explained by a Random Forest model trained by region is 41.8%, 43.9%, and 46.8% for the south, central and north regions (results not shown). This indicates that the capacity of the predictor variables to describe variations in EONR slightly increases when moving north. Of interest, corn land is at a higher percentage in the north and the cropping system is mostly corn-soy. Archontoulis et al. (2020) also found their prediction model to perform better in high yielding environments (e.g., north Illinois) compared to low yielding environments. They argue that the reason is that their model did not account for all stress limitations common in low-yielding environments.

A cause for the improved accuracy in the north could be that, in that region, a large proportion of the EONR variation is explained by the soil supply side of the N cycle, mainly by SOM mineralization and soil N concentration (Fig. 1d). This can be supported by observing the ratio between crop N uptake when no fertilizer was applied, which represents the amount of N provided by soil processes (including initial N and SOM mineralization), and crop N uptake at $EONR^{ex\text{-}post}$, which represents the amount of N that the crop needs to maximize profits. This ratio is 25%, 41%, and 55% for the south, central and north regions (results not shown). Since the model knows soil N and SOM at v5, it can provide more accurate predictions. On the other hand, in areas with lower SOM, the EONR variation depends more on crop N demand, which is driven by unknown season's growing conditions, and less by known soil characteristics. The variable importance plots for the three different random forest models trained for each region (Fig. S3) show that the two variables that represent the soil supply side of the N cycle escalate in the ranking when moving north. Initially, soil N moves from third to first place when moving from south to the central region, and organic matter becomes the first and soil N third in the north.

The second reason the dynamic value was higher when moving north is higher intra-region variability in the amount of N provided by the soil. Since static recommendations maximize profits across most of the growing conditions in a region, it is expected that the dynamic value will be higher on fields with conditions that are uncommon to their region. We analyzed the relationship between dynamic value and SOM (Fig. 5a) and dynamic value and crop N uptake when no fertilizer was applied (Fig. 5b). In fields far below or above the average conditions, the dynamic value was higher (seen in the U-shaped dynamic value curve, with a higher dynamic value on the extremes and zero dynamic value in the center). This relationship becomes more evident when moving north because there is an increase of intra-regional variation in the soil's capacity to provide N, driven by higher SOM variability. This provides an opportunity for improving both static and dynamic tools. Dynamic tools could be targeted to fields that are far from the average SOM content in the area to increase the likelihood of producing value. Likewise, static

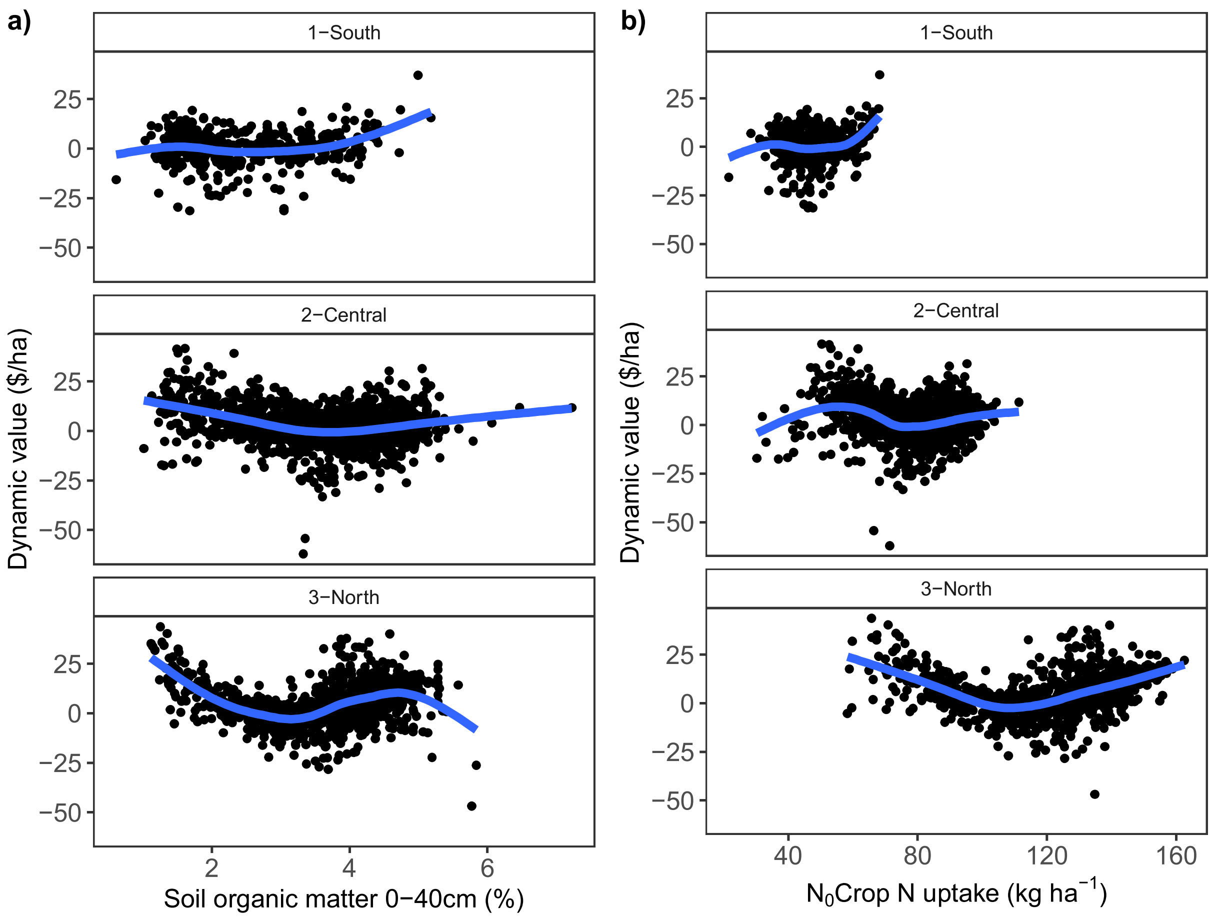

Fig. 5. (a) Dynamic value by soil organic matter (om_40cm). (b) Dynamic value by crop N uptake with zero N. Lines represent the fitted regression, using the "Locally Weighted Least Squares Regression" method (loess). *Source*: 4030 evaluation fields, aggregated for the 30 years.

tools could improve their predictions by increasing the regions' resolution, making them more homogeneous in their SOM content.

4. Discussion

Our computer modeling framework allows us to compare N recommendation tools at a geographic scale and time period that has not been done before. This framework captured net differences over millions of aggregated scenarios, providing new insights into the conflict between economic and environmental sustainability outcomes associated with N management in maize production. Compared to previous work that accounted for a limited number of locations, our conclusions are robust, not being easily misled by the results in individual fields or particular weather-years.

Focusing on the realistic NRTs (excluding the ex_post and rf_future), our results highlight that profits did not change significantly with the chosen tool. The difference between the most profitable (rf_full) and the least profitable tool (rf_reduced) was only 8 $/ha at the state level (Table 2). Moreover, the rf_full, a highly complex tool involving machine learning and precise weather forecasts, only increased profits by 2 $/ha compared with the simpler mrtn_peak. Considering the uncertainty and variability associated with farm production, such differences are relatively small, and presumably, farmers would prefer to spend their time and effort optimizing other decisions that will have a higher pay-off (Pannell, 2006). Previous research has also found a lack of better performance by complex tools (Clark et al., 2020; Qin et al., 2018; Puntel et al., 2018; Ransom et al., 2020). Together these results question a major assumption in this field, i.e., that accounting for real-time soil and crop conditions with dynamic N management strategies increases profitability. A strength of our framework is that it supported a detailed investigation into why dynamic tools could not provide high and consistent value for farmers compared to simple long-term static recommendations.

A simple starting point to understand why more accurate tools did not increase profits is often overlooked in previous studies. In the profits curve, the economic penalty of recommendations below the $EONR^{ex\text{-}post}$ is higher than above (Fig. 3). The reason is in the shape of the yield response curve, where rapid yield gains occur before a plateau is reached at optimal N rates, and then with N rates beyond this, the economic penalty is only the extra cost of N, which is lower than the yield penalty. Considering the high variability of the EONR (Fig. 2), with a clear year effect usually unknown beforehand, illustrates how challenging it is to provide accurate N recommendations without sometimes falling below the $EONR^{ex\text{-}post}$ and paying that higher economic penalty.

The best static tool, the mrtn_peak, is based on the MRTN methodology, designed to maximize long-term profits for a broad set of fields and weather conditions. In this attempt, profits for each N rate are averaged over multiple trials, and the N rate that maximizes those averaged profits is selected. Mathematically, trials with a higher response of profits to N have a higher weight on the final recommendations. Similarly, trials with a null response of profits to N do not influence the MRTN rate. As a consequence, MRTN provides recommendations that are higher than the mean EONR across sites -9 kg N/ha on average, 56% of the times (Table 2). This allows the tool to maintain high profits since it recommends N rates below $EONR^{ex\text{-}post}$ less often, avoiding the costly economic penalty resulting from grain yield reductions.

Dynamic models are based on the idea that higher accuracy will increase profits. The analysis of our rf_full showed that in the attempt to predict the EONR better, it necessarily falls more often below the $EONR^{ex\text{-}post}$ than the mrtn_peak (Fig. 4b). This creates an uneven game since being more accurate means being exposed to higher economic penalties. This lower N rate recommendation is warranted in some cases, meaning the rf_full tool provides a better prediction. However, predictions are subject to uncertainty, and other times rf_full recommend a lower N rate than the mrtn_peak, and it fails, lowering the final profits.

A significant finding is that even under these conditions when the rf_full tool succeeds to outperform the mrtn_peak tool, the amount of the value gained is smaller than the amount lost when it fails (Fig. 4a). In other words, the savings associated with lower fertilizer costs do not compensate for the lost revenue due to slight decreases in yield. In consequence, even if the rf_full tool is more often successful (Fig. 4b), the final dynamic value produced across thousands of fields can still be negative or very small (Fig. 4c). For example, rf_full was successful in 46%, neutral in 22%, and failed in 33% of cases, but the overall dynamic value was only 2 $/ha. To our knowledge, our simulation analysis is the first to demonstrate how these simple mathematical relationships represent a major challenge for any dynamic strategy. While many efforts are dedicated to improving the performance of dynamic tools (Sela et al., 2016; Puntel et al., 2018; Qin et al., 2018; Kitchen et al., 2010) we caution that extremely high precision in dynamic recommendations would be needed for overcoming the difference in the economic outcome. Nevertheless, based on previous work, this is unlikely to be achieved due to the lack of predictability of the complex spatiotemporal interactions between soil properties, weather conditions, and genetics, which collectively determine crop response to N fertilizer input (Tremblay et al., 2012; Sela et al., 2016; Morris et al., 2018).

An assessment of the conditions under which rf_full recommendations failed in our study illustrates the complexity of this challenge. When profits were added for each field over the 30 weather-years, even if the rf_full recommendations provided value in a region, there are still a high proportion of fields (46%, 42%, 34% for south, central and north region) for which profits are higher when using the mrtn_peak tool (Table S2). This reinforces the idea that it will be difficult to convince farmers to adopt a complex tool when there are inconsistent benefits, and it is challenging to know beforehand which method will perform better in their fields in the long term.

In our study area, we found conditions that made the rf_full tool perform more consistently, and it translated into slightly higher dynamic value. These conditions occur in the north, and they were a more beneficial dynamic value per outcome (i.e., when inaccurately predicting below MRTN, the profits penalty was lower) (Fig. 4a), combined with higher accuracy of rf_full predictions (i.e., greater frequency of success vs. failure across bins). We hypothesize that the driver of these outcomes is higher SOM, which makes the profits curve flatter (lowering the yield penalty for low recommendations) and increases the predictability of the EONR (because it depends more on soil N and SOM, which are predictor variables of the tool, and less on random weather conditions). Moreover, in the north and central regions, fields with SOM far from the average had a positive dynamic value, showing that the rf_full overperform mrtn_peak when a field's SOM is not representative of the region it belongs (Fig. 5). Soil N supply is essential in regulating both yield response and the need for additional N fertilizer; hence a large amount of research is focused on incorporating soil N supply into N management approaches (Bundy and Andraski, 1995; Meisinger et al., 2008; Clark et al., 2020; Ransom et al., 2021). Nevertheless, several factors change when moving north in our study, and we cannot prove causation. This finding provides conditions where dynamic tools could perform better, and further research is needed to validate these ideas.

It is noteworthy that rf_full, trained with thousands of trials in a perfectly controlled simulation environment, failed to achieve an accuracy high enough to provide consistent dynamic value over static recommendations. Therefore, we argue that it will be even more difficult to achieve such accuracy by tools working with real data. Our Random Forest model is the result of multiple comparisons of models (XBoost, CNN, boosted regression – results not shown), and it was the one that best predicted the EONR. It is built with a huge amount of trials, far from what is attainable by current on-farm or on-station research platforms in the sector. It also includes seventeen predictor variables, measured on each field and selected for their capacity to improve predictions. Consequently, we believe that the accuracy obtained in this study with dynamic tools is more than what any other tool will achieve in real-

world conditions. For instance, our rf_full achieved an RMSE of 46 kg/ha (Table 2), while a study that evaluated 31 NRTs over 49 sites in real conditions reported RMSE ranging from 70 to 122 kg/ha (Ransom et al., 2020). That being said, many dynamic NRTs are being developed, and they should be tested to address this challenge. We have applied machine learning, but this is fundamentally different from other dynamic simulation approaches using process-based crop models like APSIM or Adapt-N (Puntel et al., 2018; Sela et al., 2016) and many improvements could occur in the field of digital agriculture that could increase the accuracy of dynamic tools. One promising area to increase the dynamic value is incorporating additional factors like plant population and genetics that interact with N response instead of only providing N recommendations.

The value of NRTs can also be assessed in terms of reduced N leaching as an additional criterion. Profitability is the most important consideration of farmers, and it likely is a current barrier to adoption. Nevertheless, environmental aspects are likely the main interest of regulatory bodies, and indicators such as N leaching will become more relevant going forward. Given the context of maize production covering such a large area in the US Midwest, changes of small increments in N leaching can add up to substantial reductions for the region. To more effectively address N pollution, many scientists are calling for profitability and environmental footprint to have similar weightage in N management decisions (Sobota et al., 2015; Jin et al., 2019).

When looking at environmental results, there are two main insights obtained from our analysis. The first insight is that there is a potential for N leaching reductions when farmers are using NRTs that recommend N rates that are too high. Surveys in the area show that 67% farmers in the region are applying N rates closer to the high range of the MRTN tool (Sellars et al., 2020). If these farmers change to more eco-efficient approaches (such as mrtn_low, or rf_full), reductions of approximately 15% could be obtained without reducing profits (Table 2). Previous research has also found great potential for reductions in N leaching when comparing dynamic tools with tools that tend to overpredict N rates, like grower selected N rates (Sela et al., 2016) or yield-based recommendations tools (Sela et al., 2017).

The second insight is that both tools can achieve similar N leaching reductions, and there is no clear environmental benefit of using dynamic recommendations. The rf_full provided lower N leaching by recommending N rates with higher accuracy. Nevertheless, the MRTN tool could also support environmental goals by recommending N rates on the lower end of their range, with minimal effect on profits (Table 2).

In this matter, the MRTN description acknowledges that the response at the top of the curve is flat: “An interesting result of the MRTN analysis is that the net return to N is fairly flat at rates that surround the point of maximum net return” (Sawyer et al., 2006). Consequently, they recommend a range of rates above or below 2.47 $/ha (1 $/acre) from the top and let farmers and crop consultants choose the N rate inside that range. Even though profits are similar in that range, the environmental impact is not. Given the complexities of N dynamics, and the tendency of farmers to overapply (Vanotti and Bundy, 1994), farmers may not be able to optimize the decision in that range and will probably be biased to the high end. A safer and more eco-efficient approach for the MRTN methodology is to provide recommendations on the low end, contributing to the environmental goals without hurting profits significantly. They could also find ways to show the environmental risk to farmers to create awareness about the externalities of the decision to apply the high vs. low end of the range.

4.1. *Limitations, opportunities, and future studies*

Some considerations should be made when interpreting our findings. One is that we focused this study on the state of Illinois. The state is located in a highly productive area, with a flat landscape that leads to relatively low variation in soil types compared to other landscapes. Additionally, soils receive rain and snow during winter and are usually at full water capacity in early spring. These conditions produce a lower spatial and temporal heterogeneity than other regions, limiting our findings to the described area. The comparison of dynamic and static tools may be different in other areas.

Another consideration is that we used a calibrated crop model to generate the needed data. Using crop models has the advantage of allowing evaluations at spatial and time scales that would be cost-prohibitive to do in real situations. As a limitation of our study, we recognize that crop models are designed to represent natural processes. As with any model, they could have errors, and the output can be different from what is happening in real situations. Nevertheless, we consider that we limited those errors by calibrating the main variables that would affect the conclusion using real data. We assume that any model bias, if it exists, will be constant across tools, and relative comparisons will remain accurate (Baum et al., 2020).

Our results show that there is an opportunity for improving N recommendations. The inclusion of other factors that would affect crop N response could be incorporated into static tools. Currently, MRTN does not account for factors like planting date, genetics, plant population, or application timing. Dynamic models can incorporate these factors, potentially increasing the dynamic value. Nevertheless, in the future, MRTN could also incorporate these factors, and relative differences will remain. We also acknowledge that our economic analysis was only partial, and a full-system accounting of costs and benefits is needed. For example, MRTN is based on hundreds of field trials conducted across the Midwest each year, which has significant costs. These trials must continue to keep pace with changes in genetics, management, and weather interactions. In contrast, the dynamic tools evaluated here rely on high information, which is very costly to collect across a sufficient number of fields. Both of these factors, as well as changes in N application equipment, fertilizer source, decision-support tool development (e.g. dynamic tool app), environmental damage costs of N leaching, and other aspects should be included in future work.

In summary, considering the limitations of the approach followed in this work, results should be limited to the study area and be regarded as insights or relative values instead of exact values. Further studies could assess the insights found in this work with actual data and include more factors that affect N response.

5. Conclusion

Our work provided actionable insights, with comparisons of several groups of dynamic and static NRTs across space and time to understand their impacts on economic and environmental performance. From a practical view, our assessment of dynamic and static tools shows they do not differ significantly in their profits, suggesting that there is limited opportunity to increase adoption based solely on a promise of higher economic performance if consistently implemented over many years. Nevertheless, the tools differ in their environmental performance, and there is an opportunity to improve current tools in this matter, resulting in a 15% reduction in N leaching. Static tools, like MRTN, can do it in a simpler way, reducing recommendation over the whole area to the lower end of the flat part of the profits curve, reducing N leaching with low harm to farmers’ profits. Dynamic tools, like our theoretical rf_full, reduce N leaching due to their higher accuracy and lower overall recommended N rates. Dynamic tools require more information and effort but can be better perceived by farmers by incorporating site-specific information. Unfortunately, our results suggest there are little economic incentives at the state level that would induce farmers to adopt any of these more eco-efficient tools since they will not improve their finances in the long run. This dilemma presents a key opportunity for stakeholders and policymakers to design policies that provide those incentives and take advantage of data-driven solutions to lower the environmental impact of our agriculture.

Declaration of Competing Interest

The authors declare no conflict of interest that could have appeared to influence the work reported in this paper.

Acknowledgement

This study was conducted thanks to the support of NIFA Hatch \Multistate Hatch Grant, Enhancing nitrogen utilization in corn based cropping systems to increase yield, improve profitability and minimize environmental impacts, ILLU-802-965.

Appendix A. Supplementary data

Supplementary data associated with this article can be found, in the online version, at https://doi.org/10.1016/j.agsy.2021.103275.

References

Archontoulis, S.V., Castellano, M.J., Licht, M.A., Nichols, V., Baum, M., Huber, I., Martinez-Feria, R., Puntel, L., Ordóñez, R.A., Iqbal, J., et al., 2020. Predicting crop yields and soil-plant nitrogen dynamics in the US corn belt. Crop Sci. 60 (2), 721–738.
Banger, K., Nafziger, E.D., Wang, J., Muhammad, U., Pittelkow, C.M., 2018. Simulating nitrogen management impacts on maize production in the US Midwest. PLoS One 13 (10), e0201825.
Basche, A.D., Archontoulis, S.V., Kaspar, T.C., Jaynes, D.B., Parkin, T.B., Miguez, F.E., 2016. Simulating long-term impacts of cover crops and climate change on crop production and environmental outcomes in the Midwestern United States. Agric. Ecosyst. Environ. 218, 95–106.
Basso, B., Shuai, G., Zhang, J., Robertson, G.P., 2019. Yield stability analysis reveals sources of large-scale nitrogen loss from the US Midwest. Sci. Rep. 9 (1), 1–9.
Bates, D., Mächler, M., Bolker, B., Walker, S., 2015. Fitting linear mixed-effects models using LME4. J. Stat. Softw. 67 (1), 1–48.
Baum, M.E., Licht, M.A., Huber, I., Archontoulis, S.V., 2020. Impacts of climate change on the optimum planting date of different maize cultivars in the central US corn belt. Eur. J. Agron. 119, 126101.
Boryan, C., Yang, Z., Mueller, R., Craig, M., 2011. Monitoring us agriculture: the US department of agriculture, national agricultural statistics service, cropland data layer program. Geocarto Int. 26 (5), 341–358.
Breiman, L., 2001. Random forests. Mach. Learn. 45, 1.
Bundy, L., Andraski, T., 1995. Soil yield potential effects on performance of soil nitrate tests. J. Prod. Agric. 8 (4), 561–568.
Clark, J.D., Fernández, F.G., Veum, K.S., Camberato, J.J., Carter, P.R., Ferguson, R.B., Franzen, D.W., Kaiser, D.E., Kitchen, N.R., Laboski, C.A., et al., 2020. Soil-nitrogen, potentially mineralizable-nitrogen, and field condition information marginally improves corn nitrogen management. Agronomy J. 112 (5), 4332–4343.
Correndo, A.A., Rotundo, J.L., Tremblay, N., Archontoulis, S., Coulter, J.A., Ruiz-Diaz, D., Franzen, D., Franzluebbers, A.J., Nafziger, E., Schwalbert, R., et al., 2020. Assessing the uncertainty of maize yield without nitrogen fertilization. Field Crops Res. 260, 107985.
Finger, R., 2012. Nitrogen use and the effects of nitrogen taxation under consideration of production and price risks. Agric. Syst. 107, 13–20.
Holzworth, D., Huth, N., Devoil, P., Zurcher, E., Herrmann, N., Mclean, G., Chenu, K., Van Oosterom, E., Snow, V., Murphy, C., Moore, A., Brown, H., Whish, J., Verrall, S., Fainges, J., Bell, L., Peake, A., Poulton, P., Hochman, Z., Thorburn, P., Gaydon, D., Dalgliesh, N., Rodriguez, D., Cox, H., Chapman, S., Doherty, A., Teixeira, E., Sharp, J., Cichota, R., Vogeler, I., Li, F., Wang, E., Hammer, G., Robertson, M., Dimes, J., Whitbread, A., Hunt, J., Van Rees, H., Mcclelland, T., Carberry, P., Hargreaves, J., Macleod, N., Mcdonald, C., Harsdorf, J., Wedgwood, S., Keating, B., 2014. APSIM – evolution towards a new generation of agricultural systems simulation. Environ. Model. Softw. 62, 327–350.
Iqbal, J., Necpalova, M., Archontoulis, S.V., Anex, R.P., Bourguignon, M., Herzmann, D., Mitchell, D.C., Sawyer, J.E., Zhu, Q., Castellano, M.J., 2018. Extreme weather-year sequences have nonadditive effects on environmental nitrogen losses. Global Change Biol. 24 (1), e303–e317.
Jin, Z., Ainsworth, E.A., Leakey, A.D., Lobell, D.B., 2018. Increasing drought and diminishing benefits of elevated carbon dioxide for soybean yields across the US Midwest. Global Change Biol. 24 (2), e522–e533.
Jin, Z., Archontoulis, S.V., Lobell, D.B., 2019. How much will precision nitrogen management pay off? An evaluation based on simulating thousands of corn fields over the US corn-belt. Field Crops Res. 240, 12–22.
Kitchen, N.R., Sudduth, K.A., Drummond, S.T., Scharf, P.C., Palm, H.L., Roberts, D.F., Vories, E.D., 2010. Ground-based canopy reflectance sensing for variable-rate nitrogen corn fertilization. Agronomy J. 102 (1), 71–84.
Kuhn, A., Gaiser, T., Gandonou, E., 2010. Simulating the effects of tax exemptions on fertiliser use in benin by linking biophysical and economic models. Agric. Syst. 103 (8), 509–520.
Lawes, R.A., Oliver, Y.M., Huth, N.I., 2019. Optimal nitrogen rate can be predicted using average yield and estimates of soil water and leaf nitrogen with infield experimentation. Agronomy J. 111 (3), 1155–1164.
Liaw, A., Wiener, M., 2002. Classification and regression by randomforest. R News 2 (3), 18–22. https://CRAN.R-project.org/doc/Rnews/.
Lory, J., Scharf, P., 2003. Yield goal versus delta yield for predicting fertilizer nitrogen need in corn. Agronomy J. 95 (4), 994–999.
Mandrini, G., Pittelkow, C.M., Archontoulis, S.V., Martin, N.F., 2021. Simulations of corn response to nitrogen over thousands of fields and multiple years in Illinois. Data in Brief.
McNunn, G., Heaton, E., Archontoulis, S., Licht, M., VanLoocke, A., 2019. Using a crop modeling framework for precision cost-benefit analysis of variable seeding and nitrogen application rates. Front. Sustainable Food Syst. 3, 108.
Meisinger, J., 1984. Evaluating plant-available nitrogen in soil-crop systems. Nitrogen Crop Prod. 389–416.
Meisinger, J.J., Schepers, J., Raun, W., 2008. Crop nitrogen requirement and fertilization. Nitrogen Agric. Syst. 49, 563–612.
Millar, N., Robertson, G.P., Grace, P.R., Gehl, R.J., Hoben, J.P., 2010. Nitrogen fertilizer management for nitrous oxide (N2O) mitigation in intensive corn (maize) production: an emissions reduction protocol for US Midwest agriculture. Mitig. Adapt. Strateg. Global Change 15 (2), 185–204.
Morris, T.F., Murrell, T.S., Beegle, D.B., Camberato, J.J., Ferguson, R.B., Grove, J., Ketterings, Q., Kyveryga, P.M., Laboski, C.A., McGrath, J.M., et al., 2018. Strengths and limitations of nitrogen rate recommendations for corn and opportunities for improvement. Agronomy J. 110 (1), 1–37.
Natural Resources Conservation Service, U. S. D. o. A., 2018. Soil Survey Geographic (SSURGO) Database for Illinois accessed online.
Pannell, D.J., 2006. Flat earth economics: the far-reaching consequences of flat payoff functions in economic decision making. Rev. Agric. Econ. 28 (4), 553–566.
Pasley, H.R., Virginia, N., Castellano, M.J., Helmers, M.J., Baum, M.E., Kladivko, E.J., Archontoulis, S., 2021. Rotating maize reduces the risk and rate of nitrate leaching. Environ. Res. Lett.
Puntel, L.A., Sawyer, J.E., Barker, D.W., Dietzel, R., Poffenbarger, H., Castellano, M.J., Moore, K.J., Thorburn, P., Archontoulis, S.V., 2016. Modeling long-term corn yield response to nitrogen rate and crop rotation. Front. Plant Sci. 7, 1630.
Puntel, L.A., Sawyer, J.E., Barker, D.W., Thorburn, P.J., Castellano, M.J., Moore, K.J., VanLoocke, A., Heaton, E.A., Archontoulis, S.V., 2018. A systems modeling approach to forecast corn economic optimum nitrogen rate. Front. Plant Sci. 9, 436.
Qin, Z., Myers, D.B., Ransom, C.J., Kitchen, N.R., Liang, S.-Z., Camberato, J.J., Carter, P. R., Ferguson, R.B., Fernandez, F.G., Franzen, D.W., et al., 2018. Application of machine learning methodologies for predicting corn economic optimal nitrogen rate. Agronomy J. 110 (6), 2596–2607.
Ransom, C.J., Kitchen, N.R., Camberato, J.J., Carter, P.R., Ferguson, R.B., Fernández, F. G., Franzen, D.W., Laboski, C.A., Nafziger, E.D., Sawyer, J.E., et al., 2020. Corn nitrogen rate recommendation tools' performance across eight US Midwest corn belt states. Agronomy J. 112 (1), 470–492.
Ransom, C.J., Kitchen, N.R., Sawyer, J.E., Camberato, J.J., Carter, P.R., Ferguson, R.B., Fernández, F.G., Franzen, D.W., Laboski, C.A., Myers, D.B., et al., 2021. Improving publicly available corn nitrogen rate recommendation tools with soil and weather measurements. Agronomy J.
Sawyer, J., Nafziger, E., Randall, G., Bundy, L., Rehm, G., Joern, B., et al., 2006. Concepts and Rationale for Regional Nitrogen Rate Guidelines for Corn. Iowa State University-University Extension, Ames, Iowa.
Scharf, P., 2015. Managing Nitrogen in Crop Production. Wiley Online Library.
Scharf, P.C., Brouder, S.M., Hoeft, R.G., 2006. Chlorophyll meter readings can predict nitrogen need and yield response of corn in the north-central USA. Agronomy J. 98 (3), 655–665.
Schmidt, J.P., Dellinger, A.E., Beegle, D.B., 2009. Nitrogen recommendations for corn: an on-the-go sensor compared with current recommendation methods. Agronomy J. 101 (4), 916–924.
Sela, S., van Es, H., Moebius-Clune, B., Marjerison, R., Kneubuhler, G., 2018. Dynamic model-based recommendations increase the precision and sustainability of N fertilization in Midwestern US maize production. Comput. Electron. Agric. 153, 256–265.
Sela, S., Van Es, H., Moebius-Clune, B., Marjerison, R., Melkonian, J., Moebius-Clune, D., Schindelbeck, R., Gomes, S., 2016. Adapt-N outperforms grower-selected nitrogen rates in northeast and Midwestern United States strip trials. Agronomy J. 108 (4), 1726–1734.
Sela, S., van Es, H.M., Moebius-Clune, B.N., Marjerison, R., Moebius-Clune, D., Schindelbeck, R., Severson, K., Young, E., 2017. Dynamic model improves agronomic and environmental outcomes for maize nitrogen management over static approach. J. Environ. Qual. 46 (2), 311–319.
Sellars, S.C., Schnitkey, G.D., Gentry, L.F., 2020. Do Illinois Farmers Follow University-Based Nitrogen Recommendations?.
Semaan, J., Flichman, G., Scardigno, A., Steduto, P., 2007. Analysis of nitrate pollution control policies in the irrigated agriculture of Apulia region (Southern Italy): a bio-economic modelling approach. Agric. Syst. 94 (2), 357–367.
Shahhosseini, M., Martinez-Feria, R.A., Hu, G., Archontoulis, S.V., 2019. Maize yield and nitrate loss prediction with machine learning algorithms. Environ. Res. Lett. 14 (12), 124026.
Sobota, D.J., Compton, J.E., McCrackin, M.L., Singh, S., 2015. Cost of reactive nitrogen release from human activities to the environment in the United States. Environ. Res. Lett. 10 (2), 025006.
Thornton, P.E., Thornton, M.M., Mayer, B.W., Wilhelmi, N., Wei, Y., Devarakonda, R., Cook, R.B., 2014. Daymet: Daily Surface Weather Data on a 1-km Grid for North

America, Version 2. Tech. Rep. Oak Ridge National Lab. (ORNL), Oak Ridge, TN. (United States).

Tremblay, N., Bouroubi, Y.M., Bélec, C., Mullen, R.W., Kitchen, N.R., Thomason, W.E., Ebelhar, S., Mengel, D.B., Raun, W.R., Francis, D.D., et al., 2012. Corn response to nitrogen is influenced by soil texture and weather. Agronomy J. 104 (6), 1658–1671.

Vanotti, M., Bundy, L., 1994. An alternative rationale for corn nitrogen fertilizer recommendations. J. Prod. Agric. 7 (2), 243–249.

Vitousek, P.M., Aber, J.D., Howarth, R.W., Likens, G.E., Matson, P.A., Schindler, D.W., Schlesinger, W.H., Tilman, D.G., 1997. Human alteration of the global nitrogen cycle: sources and consequences. Ecol. Appl. 7 (3), 737–750.

Zhu, Q., Schmidt, J., Lin, H., Sripada, R., 2009. Hydropedological processes and their implications for nitrogen availability to corn. Geoderma 154 (1–2), 111–122.
